# BiCyCLE NMES—neuromuscular electrical stimulation in the perioperative treatment of sarcopenia and myosteatosis in advanced rectal cancer patients: design and methodology of a phase II randomised controlled trial

**DOI:** 10.1186/s13063-021-05573-2

**Published:** 2021-09-15

**Authors:** Edward T. Pring, Laura E. Gould, George Malietzis, Philip Lung, Mina Bharal, Tutu Fadodun, Paul Bassett, Mani Naghibi, Claire Taylor, Ioanna Drami, Deeptika Chauhan, Tamsyn Street, Nader K. Francis, Thanos Athanasiou, John M. Saxton, John T. Jenkins, on behalf of the BiCyCLE Research Group

**Affiliations:** 1 George Davies Research Fellowship, St Mark’s Hospital, Harrow, UK; 2 Complex Cancer Clinic, St Mark’s Hospital, Watford Road, Harrow, HA1 3UJ UK; 3 Department of Surgery and Cancer, Imperial College, London, W2 1NY UK; 4 Department of Surgery, St. Mark’s Hospital, Watford Road, Harrow, Middlesex, HA1 3UJ UK; 5 College of Medical Veterinary and Life Sciences, University of Glasgow, Glasgow, G12 8QQ UK; 6 Statsconsultancy Ltd, Amersham, Bucks HP7 9EN UK; 7 Department of Clinical Science and Engineering, Salisbury District Hospital, Salisbury, UK; 8 Department of Surgery, Yeovil District Hospital, Higher Kingston, Yeovil, BA21 4AT UK; 9 Department of Sport, Exercise and Rehabilitation, Faculty of Health & Life Sciences, Northumbria University, Newcastle Upon Tyne, NE1 8ST UK

**Keywords:** Advanced rectal cancer, Sarcopenia, Myosteatosis, Rehabilitation, Exenteration surgery, NMES, Neuromuscular electrical stimulation

## Abstract

**Background:**

Colorectal cancer is associated with secondary sarcopenia (muscle loss) and myosteatosis (fatty infiltration of muscle) and patients who exhibit these host characteristics have poorer outcomes following surgery. Furthermore, patients, who undergo curative advanced rectal cancer surgery such as pelvic exenteration, are at risk of skeletal muscle loss due to immobility, malnutrition and a post-surgical catabolic state. Neuromuscular electrical stimulation (NMES) may be a feasible adjunctive treatment to help ameliorate these adverse side-effects. Hence, the purpose of this study is to investigate NMES as an adjunctive pre- and post-operative treatment for rectal cancer patients in the radical pelvic surgery setting and to provide early indicative evidence of efficacy in relation to key health outcomes.

**Method:**

In a phase II, double-blind, randomised controlled study, 58 patients will be recruited and randomised (1:1) to either a treatment (NMES plus standard care) or placebo (sham-NMES plus standard care) group. The intervention will begin 2 weeks pre-operatively and continue for 8 weeks after exenterative surgery. The primary outcome will be change in mean skeletal muscle attenuation, a surrogate marker of myosteatosis. Sarcopenia, quality of life, inflammatory status and cancer specific outcomes will also be assessed.

**Discussion:**

This phase II randomised controlled trial will provide important preliminary evidence of the potential for this adjunctive treatment. It will provide guidance on subsequent development of phase 3 studies on the clinical benefit of NMES for rectal cancer patients in the radical pelvic surgery setting.

**Trial registration:**

Protocol version 6.0; 05/06/20. ClinicalTrials.gov NCT04065984. Registered on 22 August 2019; recruiting.

**Supplementary Information:**

The online version contains supplementary material available at 10.1186/s13063-021-05573-2.

## Background

Radical multi-visceral resection of pelvic tumours, known as pelvic exenteration, is being utilised to successfully treat a number of intra-abdominal malignancies [1]. Pelvic exenteration for locally recurrent (LRRC) or primary advanced rectal cancer has a high morbidity and mortality. The *PelvEx Collaborative* analysed data from 1184 patients, who underwent surgery for LRRC, and found that 2% of patients died within 30 days of surgery and 32% of patients experienced a major complication [2]. Despite this high morbidity and mortality, these complex procedures are increasingly practised in specialist centres. Following surgery, these patients enter a catabolic crisis where incapacitation and high protein and fat metabolism lead to a marked loss in skeletal muscle [3–5]. Low skeletal muscle mass (sarcopenia) and fatty infiltration (myosteatosis) are independently associated with poorer post-operative outcomes following surgery for colorectal cancer [6, 7]. The aetiology behind sarcopenia and myosteatosis is complex and multifactorial and includes inflammatory changes, hormonal changes, loss of function, fatigue and energy balance [8]. However, strategies to preserve skeletal muscle mass, quality and function may improve these outcomes.

A meta-analysis of resistance exercise training in patients with non-metastatic cancer showed significantly increased skeletal muscle mass [9]. Exercise can also impart an anti-inflammatory effect by attenuating the cellular response to inflammatory stimuli and pro-inflammatory cytokines such as IL-6, TNFα and TGFβ [10, 11]. However, exercise programmes following a rectal cancer diagnosis and exenterative surgery are not always possible or practical due to patient anxieties and the need to expedite treatment, and the pain or disability associated with the extensiveness of the surgery itself. Furthermore, restriction upon rehabilitative resources, especially physiotherapy, often leaves patients immobile for long periods with resultant muscle atrophy. An alternative approach to traditional physiotherapy could be functional electrical stimulation (FES) via neuromuscular electrical stimulation (NMES). This is currently used in clinical practice for a number of diseases, indeed, at the National Clinical FES Centre at Salisbury, UK, over 2500 patients are currently undergoing FES [12]. NMES of the lower-limb muscles requires less motivation than traditional exercise and can be undertaken whilst the patient is seated or lying down [13]. NMES can be used to produce a muscle contraction equivalent to 20 to 40% of a maximum voluntary contraction [14] thus meeting the criteria of the American College of Sports medicine definition of planned exercise [15].

A study of anterior cruciate ligament [of the knee] (ACL) reconstruction patients by Hasegawa and colleague demonstrated NMES, implemented during the early rehabilitation stage, was effective in maintaining and increasing muscle thickness and strength in the operated limb [16]. There is also evidence from meta-analyses that NMES increases muscle strength and shows potential benefit for joint range of motion, muscle atrophy, outcomes of ventilation and activity limitations in critically ill patients [17]. A Cochrane review of NMES in a number of diseases that cause cachexia (muscle and fat loss secondary to disease) such as COPD (Chronic obstructive pulmonary disease), CCF (Congestive cardiac failure), HIV/AIDS and cancer, suggested that NMES may be an effective treatment for muscle weakness in adults with advanced progressive disease, and could be considered as a treatment within rehabilitation programmes [18]. Two studies, a phase 2 randomised trial and its pilot study, investigated NMES in cancer cachexia. Both studies were conducted in patients with non-small cell lung cancer receiving palliative chemotherapy [13, 19]. The pilot study [19] demonstrated positive results however, in the phase 2 study of 49 patients, in which 30 were randomised to NMES, there were no significant differences in quadriceps muscle strength, thigh lean mass or physical activity level between groups [13]. The study team did however recommend further NMES studies in patients with cancer in other settings. Notably these two studies by Maddocks’ and colleagues focussed on palliative lung cancer patients; a surgical complex rectal cancer cohort is fundamentally different both by virtue of the impact of the insult of surgery on muscle but also the radical or curative nature of the treatment. The differences between these studies by Maddocks et al. and our study are summarised in Table 1.

**Table 1:** Differences between BiCyCLE NMES and earlier studies by Maddocks et al.

BiCyCLE NMES study population	Maddock’s study population
Post-operative “tumour free”	Active cancer
Confined to bed rest	Active and mobile population
Intensive inpatient support	Outpatient community care
Aiming for recovery up to or beyond baseline	Palliative and functionally declining population

Previous studies have examined NMES is the palliative setting, with inconclusive conclusions regarding efficacy [13, 19]. No work has yet been done on clinical outcomes of NMES use in colorectal cancer nor any trial in the post-operative setting for cancer surgery. A phase II trial is required to determine whether there is evidence of a potential benefit prior to justifying a phase III study. The previous studies performed in cancer patients have not examined the relationship to the systemic inflammatory response, nor has there been an assessment of immediate post-operative outcomes. Our study aims to provide evidence of early indicative evidence of efficacy in relation to key health outcomes, including skeletal muscle mass and quality (myosteatosis), markers of systemic inflammation and post-operative recovery outcomes in rectal cancer patients undergoing radical pelvic surgery.

## Study design, methods and analysis

The study is being undertaken at St Mark’s Hospital, the sponsor in London North West University Healthcare (LNWH) NHS Trust. Details of the study sponsor and their roles and responsibility including auditing, indemnity and monitoring can be found in the full trial protocol provided as supplementary material, (page VIII and pages 24-26). Detailed information relating to the trial authors, management teams and committees including their roles and responsibilities can be found in the supplementary material full trial protocol under the relevant headings. Any amendments will be processed and communicated through the NHS Health Regulation Authority, Queens Square Research and Ethics committee and LNWH Research and Development Department, see Full Protocol pages 24–25 for further details.

Patients will be blinded as to which trial arm they enter and a sham protocol will be used by the control arm. Body composition analysis of the images will be done by automated software used by an assessor blinded to the intervention to remove operator or interpretation bias.

Our aim is to compare the effect on muscle of therapeutic NMES and current best practice against placebo NMES and current best practice alone in patients undergoing advanced radical surgery for complex rectal cancer.

### Outcomes

#### Primary outcome

The difference in mean muscle attenuation (MA), of all skeletal muscle groups captured on the axial CT image at the level of the third lumbar vertebrate (L3), measured in Hounsfield units and hence the degree of myosteatosis between the pre-operative and 6-month post-operative CT scan in the NMES treatment group and the placebo NMES group.

#### Secondary outcomes

Our main secondary outcomes include change in total skeletal muscle cross-sectional area, at the L3 level between treatment and non-treatment groups as well as between time points for individual patients. Difference in quality of life between groups using the validated questionnaires ED-5Q-5L & EORTC QLQ – CR29. Post-operative complications and length of hospital stay between both arms and comparison of the systemic inflammatory response between each group. A comprehensive list of secondary outcomes is shown in Table 2.

**Table 2:** Secondary outcomes

Domain	Specific measurement	Metric	Method of aggregation	Time point
Lumbar skeletal muscle index (LSMI)	The difference in lumbar skeletal muscle index (LSMI=height / area of skeletal muscle in cm^2^ at L3) derived from the third lumbar vertebral axial level	Change in LSMI at each time point	CT Scan; SliceOmatic software version 5.0 with ABACS L3 Plug-in automation tool	Pre-surgery 3 to 6 months post-surgery
Visceral adipose tissue (VAT) Surface area	Visceral adipose tissue area (cm^2^) derived from the third lumbar vertebral axial level	Change in VAT at each time point	CT Scan; SliceOmatic software version 5.0 with ABACS L3 Plug-in automation tool	Pre-surgery 3 to 6 months post-surgery
Systemic inflammation	C-Reactive Protein and serum albumin	Modified Glasgow Prognostic Score	Ordinal values mGPS=0 mGPS=1 mGPS=2	Preoperatively and 6 months post-surgery
Cellular immune response	Neutrophil count Lymphocyte count	Neutrophil to lymphocyte ratio (neutrophil/lymphocyte count	Clinically relevant categorical cut off values NLR<3 NLR>3	Pre-operatively and 6 months post-surgery
Post-operative complications	Any post-operative complication	Clavien-Dindo Classification	Clavien-Dindo Score 1–5	Between 0 and 90 days post-surgery
Length of hospital stay	Inpatient stay post-surgery	Days	Median length of stay	From surgery to hospital discharge
Disease-free survival	Days to reported first recurrence / death / 5 years post-surgery	Days	Kaplan-Meier survival analysis	From date of surgery to 5 years post-surgery
Overall survival	Days to death / 5 years post-surgery	Days	Kaplan-Meier survival analysis	From date of surgery to 5 years post-surgery
Quality of life (General)	EuroQol 5-level EQ-5D5L Mobility, self-care, usual activities,	Visual analogue scale score 1–100 and score in each of the 5 domains	Change in scores between events	Pre-surgery, 6 and 12 months post-surgery
	Pain/discomfort and anxiety/depression			
Quality of life (colorectal specific)	EORTC QLQ - CR29 Function and Symptoms	4 multi-item scales and 19 single items assessing common symptoms and problems in colorectal cancer	Change in score between events	Pre-surgery, 6 and 12 months post-surgery
Function	Berg Balance Scale (BBS)	Berg balance score (0–56)	Change in BBS score	Pre-surgery and 6 months post-surgery
	30 s sit to stand	Number of full sit-to stand actions completed in 30 s	Change in number of successful actions	
	6-min walk test	Distance walked in 6 minutes	Change in metres	
Thigh circumference	Thigh circumference, 5cm above the superior pole of the patella	Circumference in cm	Change in thigh circumference between groups	
Dose response to NMES	Pre- and post-operative mean muscle attenuation (L3) (MMA) and hours of device usage	Change in MMA/time	Linear regression	3–6 months post-surgery
Patient satisfaction	Free text responses and satisfaction scores derived from each domain (see patient satisfaction survey supplementary material).	Qualitative and visual analogue scale 1-10	Qualitative responses Median score between groups on each domain assessed	8 weeks post-surgery
Bioimpedance analysis	Phase angle = (Xc/R)*180°/π	Cellular resistance (R) and cellular reactance (Xc)	Change in phase angle at each time point	Baseline, day 2 post operatively, day 28 postoperatively (if in hospital), day of discharge, first post-operative follow-up appointment

#### Sample size estimation

We powered this phase II trial based upon the primary outcome. Using data from the Alberta Cancer Registry, Martin and colleagues described a standard deviation of MA at 8.6 Hounsfield units (HU) for males and 10.2 HU for females [20]. We therefore assumed an overall mean SD of 9.4 HU for both male and female patients. A difference in MA between groups of 8HU was considered to be of clinical importance, and the calculation was based on showing a difference of this size between groups.

The proposed analysis will adjust the differences at 6 months for the MA values at baseline. To allow for this approach, this adjusted is included in the sample size calculation. The size of the association between baseline and outcome MA values is relatively unknown. A fairly weak correlation of about 0.3 between the time points was assumed.

The calculations were performed using a 5% significance level and 90% power. Based on the information above, it was calculated that to show a difference in MA of 8 units between groups would require a sample size of 27 per group (54 patients in total).

To allow for an estimated dropout rate of 5%, 58 patients will be recruited into the study. The dropout rate of 5% is an estimate based on the fact that the treatment period is short and supervised for the most part in hospital. For the primary outcome to be measured, we require the pre- and post-operative CT scans and therefore anticipate a very low dropout rate due to the fact these are routine scans and the time period is relatively short. The dropout rate for the secondary outcomes may be higher as the time from surgery progresses; however, the trial is powered to the primary outcome and as such, we are not taking into account the potential drop out from the trial outside this time period encompassing the primary outcome metrics.

### Trial protocol

#### Recruitment and eligibility screening

The inclusion and exclusion criteria are shown in Table 3. Following diagnosis of locally advanced rectal cancer patients is discussed in a multidisciplinary team (MDT) meeting. Some of these patients may be felt to be suitable for radical surgery—i.e., surgery performed with the intention of a cure. If patients are deemed fit for and consent to surgery then this is performed by one of three specialist surgeons in St Mark’s Hospital, London, UK.

**Table 3:** Inclusion and exclusion criteria

Inclusion criteria	Exclusion criteria
○ Adults age 18 and above ○ Male or female ○ Primary or recurrent locally advanced rectal cancer amenable to elective radical exenterative surgery ○ ASA grades I–III ○ Able and willing to consent ○ in other concurrent trials is acceptable—following discussion with trial team of both studies	○ Lack of patient consent ○ Widespread metastases not amenable to curative resection ○ Contraindication to NMES ○ Pre-existing neuromuscular degenerative disease ○ Participation in other trials where agreement on participation not made in advance by trial teams ○ Patients with solitary colon cancer above the level of the peritoneal reflexion which does not require complex pelvic surgery

Patients who meet the inclusion criteria will be identified by the clinical team in the colorectal outpatient clinic or MDT and will then be approached by the study team with written information on the trial and given the option to enrol in the study. Consent to take part in the trial will be obtained at the next outpatient clinic appointment, which will occur in the weeks preceding surgery. Patients may be included in concomitant studies; provided agreement is obtained from each trial team. Further details regarding recruitment the strategy can be found on pages 21–22 of the Full Protocol (Supplementary Material).

Detailed information on participant withdrawal and discontinuation is provided on page 22 of the full protocol and within the consent and participant information sheet (supplementary material).

#### Randomisation

Randomisation, performed after the assessment of baseline outcomes, will take place by computer-generated randomisation software (https://www.sealedenvelope.com) on a one-to-one basis. Recruitment will be performed by the study team; randomisation will be performed by the study principal investigator. Patients who are randomised to the either arm will be blinded as to intervention and will be taught by the research team to use the stimulator; this will be at their clinic appointment following consent.

**Fig. 1:**
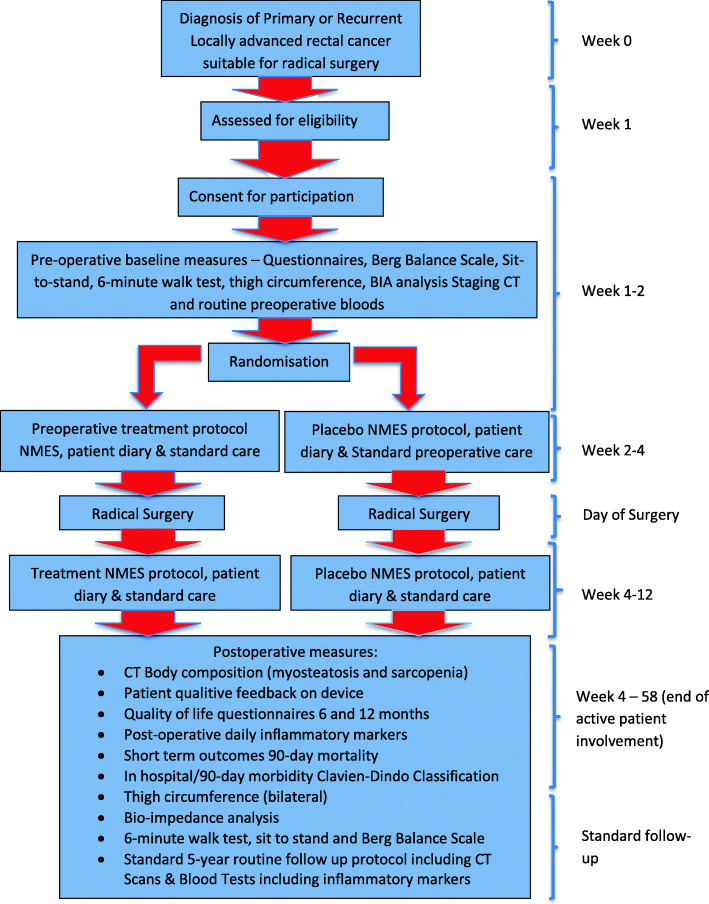
Study algorithm

**Fig. 2:**
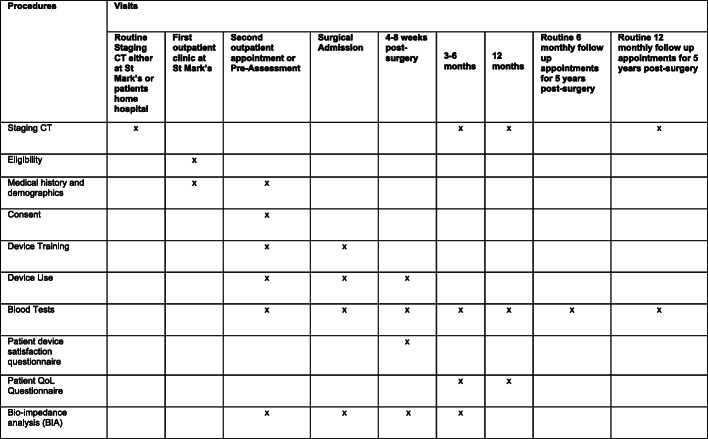
Schedule of enrolment, interventions and assessments

The NMES intervention lasts a total of 10 weeks with follow-up over 5 years. The trial algorithm is shown in Fig. 1 and schedule of enrolment, interventions and assessments in Fig. 2.

#### Blinding

Patients and the assessor of the primary outcome will be blinded. Patients will be blinded as to which arm they are in; the devices appear identical with the exception of a small, coloured plastic tab indicating whether they are treatment or placebo devices. The assessor of the primary outcome, a consultant radiologist, will be blinded as to which trial arm the participant is in; assessment of the primary outcome is also automated and therefore will not allow bias. The clinical team caring for the participant will not be aware of which trial arm the patient is in. It is not possible for the individuals providing the NMES therapy to be blinded as they will need to be aware of which arm the patient is in in order to provide effective advice.

#### Data collection

Surveillance CT scans performed as part of sequential screening (i.e. not emergency or non-routine imaging) are performed as standard in this patient group and will undergo analysis measuring mean muscle attenuation (myosteatosis) and muscle area (sarcopenia) at the level of the third lumbar vertebrate. Routine bloods including CEA (carcinoembryonic antigen) and inflammatory markers will be measured at each elective routine clinic visit and these data recorded, these samples will be collected, analysed and disposed of in accordance with the LNWH NHS Trust policy. Quality of life will be assessed at 6 and 12 months using validated quality of life questionnaires (ED-5Q-5L & EORTC QLQ – CR29). The Berg Balance scale, 30-s sit-to-stand test and 6-min walk test [21–24] will be used to assess functional outcome these tests will be performed at the patients 3 months post operatively clinic appointment. Pre- and post-operatively, we will measure bilateral thigh circumference at 15cm above the superior pole of the patella (which has been shown in earlier studies to correlate with muscle volume on MRI) [25]. Bio-impedance analysis (BIA) will be undertaken at baseline, day 2 post-operatively, day 28 post-operatively (if in hospital), day of discharge and first post-operative follow-up appointment. We will record data from the device satisfaction questionnaires from both groups. Standard outcome data and covariates to be collected are shown in Table 4.

**Table 4:** Outcomes and covariates

**Demographics** o Patient age o Patent birth year o Patient gender o Patient ethnicity coding o Weight o Height o Body mass index	**Outcome measures** o 90-day post-operative complications (Clavien-Dindo classification (18)) o Disease outcomes including 30-day survival o Death / recurrence / disease-free survival o Patient compliance diary data o Device usage
**Disease** o ASA grade (American College of Anaesthesiology) physical status o Past medical history (other or previous illnesses and surgery)	**Body composition parameters** o CT body composition at the level of the L3 vertebra o Bio-impedance analysis o Anthropometric measurements
**Cancer-specific characteristics** o Type of cancer (primary/recurrent cancer, location and subtype on histology) o Grade of cancer o Stage of cancer	**Inflammatory markers** o Serum C-reactive protein o White cell count o Serum albumin o Platelet count
**Treatment factors** o Details of surgery o Chemotherapy o Radiotherapy	**Patient-specific and functional outcomes** o Quality of life - ED-5Q-5L - EORTC QLQ – CR29 o Patient satisfaction

Data collection will be undertaken using a case report form (CRF) designed by the investigators; the data will be transferred from the CRF to a Microsoft Excel Database *(Microsoft Corporation, Redmond, Washington, USA)*, stored in the LNWH NHS Trust secure network. All collected data will be reviewed by the principal investigator prior to analysis and specific searches on written notes or electronic record systems will be made to address any missing data points. The data collected as part of this trial will be subject to review by the independent Trial Data Monitoring committee on request as per the Data Monitoring Charter (supplementary material).”

Information on data management, storage and curation is available in the Full Trial Protocol (supplementary material, page 17).

#### Data monitoring and compliance

A sponsor-approved independent data monitoring committee (IDMC) has been appointed to the trial as part of good trial governance to ensure safety, scientific validity and integrity of the trial. The data monitoring committee will have access to raw data and will review any significant adverse events or safety concerns within the trial. The IMDC will make recommendations and report directly to the sponsor representative and chief investigator. The trial Data Monitoring Charter is included for review within the supplementary material. Further information on data management and security and storage are available in the Full Protocol, supplementary material pages 17, 26 and appendix 4.

### Study intervention

#### Stimulation of muscle

We will endeavour to stimulate two major muscle groups during the study, the quadricep muscles and paraspinal muscles. The muscles of the quadriceps, particularly vastus lateralis and vastus medialis, will be stimulated in both legs; this will be performed with a view to preserving muscle mass and encouraging earlier ambulation and better function.

We will also stimulate the erector spinae muscles and the muscles of the lower back. The reason for this is twofold: firstly, it is felt that some of the earliest muscles to atrophy following surgery or during bed rest are the core muscles of the back especially as these patients will not be sitting up or utilising these important supportive muscles in the first stages of their recovery. Patients are nursed on their side during the first 14 days following major pelvic surgery, which means they tend not to use their core muscles to flex or extend their back or support their weight leading to loss. This lateral position however would afford easy access to place the electrodes. Secondly, we are focussing on the muscle groups at the third lumbar vertebrate—the level this stimulation would take place, using the device in this location, would give us the best chance of demonstrating the benefits of the device with regard to muscle preservation. This site is well away from the operative site and tumour bed in these individuals and therefore there would be no risk of stimulating the tumour bed whilst using the device in this position.

Neuro-muscular stimulation will be delivered by a *MicroStim Exercise Stimulator* MS2v2 (*Odstock Medical Limited (OML), Wiltshire, UK*) using two self-adhesive electrodes placed on the anterior thigh over the body of the vastus medialis and lateralis and the muscles of the lower back. At their second clinic appointment at St Mark’s, patients will be trained by the research fellow or other competent research team member (physiotherapist or specialist nurse) to use the NMES. A study-specific instruction leaflet will be given to this group along with the standard instruction manual by OML.

The programme will commence pre-operatively and consist of daily stimulation to one thigh at a time followed by the lower back each for 15 min, increasing to 60 min within 1 week as tolerated. One treatment session for both thighs would last between 60 and 90 min in total per day—this can be taken in up to three discrete sessions. Treatment will last for 2 weeks pre-operatively and 8 weeks postoperatively.

NMES would be used preoperatively to familiarise patients with and increase confidence in using the device prior to surgery and to aid prehabilitation.

#### Intervention training

Training in using the MicroStim 2v2 has been undertaken by the trial principal investigator at the device manufacturer, OML. Patient training will be conducted by the study PI or a trained member of their study team. Patients using the device will be observed and educated on correct usage by the study team. They will be asked to keep a usage diary and the device will record usage statistics via an inbuilt recorder. These data will feed into the analysis to provide a dose response model within the final analysis.

#### The therapeutic NMES arm

Patients will be blinded as to which arm of the trial they are in. Therapeutic NMES will be delivered by a *MicroStim Exercise Stimulator* MS2v2 using two self-adhesive electrodes placed on the anterior thigh over the body of the vastus medialis and vastus lateralis muscles and the lower back.

Pulse waveform (symmetrical biphasic squared), frequency (40 Hz), and width (350 microseconds) would be used for the duration of treatment with the NMES. The amplitude (device output 0–120 mA, tested across 1000Ω) will be set to elicit a visible and comfortable muscle contraction; patients will be encouraged to subsequently increase the amplitude as tolerated. A “compliance diary” will be kept by the patients during their treatment period detailing their time spent using the device and the settings at which they are using it.

This programme is adapted from one found to be of benefit in a pilot study of patients with non-small cell lung cancer which itself was based on an NMES exercise programme developed for patients with COPD. The stimulation parameters were selected to favour gains in function and strength over endurance (frequency), minimise skin irritation (pulse width) and allow for sufficient recovery of the muscles between contractions (duty cycle) [13, 19].

#### The placebo NMES arm

A modified model of *MicroStim Stimulator* MS2v2 (*Odstock Medical Limited, Wiltshire, UK*) will be provided to the placebo group who will apply two self-adhesive electrodes placed on the anterior thigh over the body of the vastus medialis and vastus lateralis muscles and the lower back as in the treatment group. This placebo device will be programmed to provide sub-therapeutic electrical stimulation. The device manufacturers have tailored a programme to come on and off at specified timings with ramps of specified duration. The placebo devices output is restricted to around 18V and this gives little or no muscle recruitment. Patients will however perceive a sensation of electrical stimulation.

#### Both groups

Patients in both arms will receive standard care including enhanced nutritional support (parenteral nutrition for a minimum of 5 days or until taking sufficient calories enterally) and physiotherapy in line with current guidelines and local hospital practices. Routine daily blood tests for inflammatory markers will be taken until discharge.

### Assessment of outcomes

Patients from both the treatment and control arms will receive standard 5-year follow-up. Histopathological data will be recorded following processing of the resected specimens by the pathologist. Quality of life data, patient satisfaction, bio-impedance analysis, CT Body composition and functional measurements will be taken as detailed below.

As a phase II trial, we aim to determine the short-term effects on our primary outcome; as such, our primary analysis to identify changes within the pre- and post-operative CT scans and analysis of NMES satisfaction and the initial inflammatory data, functional and quality of life data will take place at 6 months following the recruitment of the final patient with subsequent reporting and publication of these results. We will then continue long-term follow-up for the standard clinical 5-year follow-up period or until patient death. Final analysis exploring the effect of intervention on 5-year overall and disease-free survival will take place at 5 years following recruitment of the final patient.

### Body composition assessment

#### CT body composition parameters

CT image analysis using *SliceOmatic* version 5.0 software (*TomoVision, Montreal, Quebec, Canada*) will be performed. Total skeletal muscle and visceral adipose tissue surface area (cm^2^) will be evaluated on a single image at the third lumbar vertebra (L3) using HU thresholds of −29 to 150 for skeletal muscle, −50 to 150 for visceral adipose tissue (VAT) and −190 to −30 for subcutaneous adipose tissues. CT body composition analysis of all the included images will undergo automated segmentation using the ABACS L3 automated plug-in software [26] (*Voronoi Health Analytics, BC, Canada*), which complements SliceOmatic. The automated process will be directed by a radiologist who will be blinded to the treatment group of individual patients. The automated segmentation process provided by the ABACS L3 plug-in also removes the possibility of operator bias in the analysis of the images. The sum of skeletal cross-sectional muscle areas will be normalised for stature (m^2^) and reported as lumbar skeletal muscle index (LSMI) (cm^2^/m^2^). Outcome variables will be continuous; categorical variables will be defined from these data using the cut-off values described earlier [20, 27, 28].

#### Anthropometrics and bio-impedance analysis

Bio-impedance analysis (BIA) will be undertaken using a SECA mBCA 525 analyser (*SECA, Hamburg Germany*). This will be performed at baseline, day 2 post-surgery, either hospital discharge or at 28 days post-surgery (whichever is first) and at 6 months. Patients will undergo analysis in a fasted state. Posterior upper arm skin fold thickness and waist circumference will be performed at baseline and 6 months. We will measure thigh circumference at 15cm above the superior pole of the patella (which has been shown in earlier studies to correlate with muscle volume on MRI) [25]. Phase angle from BIA and patient BMI will be utilised as categorical variables with other outcome variables being continuous.

#### Functional assessment

It is important that we measure not only the anatomical effects of NMES, i.e. increased muscle mass on CT and anthropometric changes, but we identify whether these patients demonstrate both a functional and physiological improvement. To that end, we will assess functionality preoperatively at diagnosis and post-operatively at 3 months using the validated instruments of the 6-min walk test [24], 30 s sit-to-stand test [23] and Berg Balance scale (BBS) [21, 22]. These assessments will be undertaken in the complex cancer clinic by either a specialist physiotherapist or research fellow. These data will be treated as continuous outcome variables with the exception of BBS which will be categorical.

#### Quality of life

We will examine quality of life and patient experience of using the device. Quality of life will be measured using the validated questionnaires described above. On completing the intervention, participants will complete a questionnaire on compliance, comfort and usability of the device in the postoperative setting. Qualitative data and free text comments from this will also be collected.

#### Systemic inflammatory response

To monitor the inflammatory response, we will use commonly utilised postoperative inflammatory markers, namely c-reactive protein(CRP) and values derived from the full blood count and biochemistry including neutrophil-to-lymphocyte ratio (NLR) and modified Glasgow Prognostic Score (mGPS). These inflammatory markers are well-established metrics linked to both sarcopenia, myosteatosis and prognosis in colorectal cancer [29–31]. We have chosen these markers for a number of reasons; they are routinely taken and cost-effective and allow for comparison with substantial historical data. We may also require results from other trusts, due to the national spread of our patient population, and we cannot support them in obtaining non-routine tests as part of this study.

### Planned statistical analyses

This study will be performed in line with the CONSORT criteria (http://www.consort-statement.org/consort-2010). Initially outliers, patterns of attrition and missing data will be identified using a combination of graphical displays and descriptive statistics allowing decisions on the assumption of normality. The primary analysis will be of observed data only, with patients with missing data omitted from the analysis. If the primary outcome has >10% missing data points, a sensitivity analysis will be performed using multiple imputation to estimate the missing values.

Data will be analysed by a statistician blinded to the intervention. Analyses of primary and secondary endpoints will be based on the full analysis set defined according to the intention to treat principle. Safety analysis will be performed for the on all enrolled individuals with disclosure of any significant adverse events. The full analysis set consists of all participants consented and randomised with valid baseline assessments. Participants will be analysed according to the study arm they were assigned at randomisation.

The primary outcome is myosteatosis at 3–6 months post-surgery, derived from the mean muscle attenuation on CT body composition analysis. This will be analysed using analysis of covariance (ANCOVA), with muscle attenuation values at baseline used as a covariate in the analysis.

The secondary outcome measures measured on a continuous scale, and with a baseline measurement will be analysed using equivalent methods as the primary outcome. For continuous outcomes with no baseline measurement, group comparisons will be made using either the unpaired *t*-test or Mann-Whitney test, depending on data distribution. The chi-square test, or Fisher’s exact test, will be used to compare categorical outcomes between the study groups.

Significance will be assumed when *p*<0.05.

### Adverse event reporting

Adverse event reporting in this trial is carried out in accordance with the NHS Health Regulation Authority (HRA). All serious adverse events (SAE), whether or not related to participation in the trial, will be reported immediately to the trial sponsor; these will be reviewed by the trial sponsor and should further investigation be required the trial sponsor may pause the trial to carry out investigation. Should a SAE occur as a direct result of the trial device or as a direct result of participation in the trial the trial will be paused and a non-CTIMP safety report form will be submitted to the relevant research and ethics committee with 15 days of the Chief Investigator becoming aware of the event. In this instance, the SAE report will be unblinded as required by the HRA. Patient-reported adverse events deemed expected or non-serious will be reported in the final publications arising from this trial, we expect many of these minor events to be reported in the patient satisfaction questionnaire.

## Discussion

Patients who undergo major pelvic surgery have limited mobility due to postoperative pain and disability. These patients are therefore at much greater risk of suffering from muscle wasting than patients undergoing more routine colorectal surgery. This is a result of greater loss of function, greater immobility and potentially a more profound immunogenic inflammatory response.

Currently, these patients receive postoperative physiotherapy; due to limited time, postoperative pain, patient choice and resource availability, it is unlikely that the patients are exercised to their full potential. A prescribed programme with a NMES device would allow patients to choose when they undertake muscle stimulation exercise for example once they had received adequate analgesia or at a time convenient to them. This would hopefully improve compliance and bring about a hypertrophic response in the muscle.

We know that in muscle disuse in healthy individuals NMES may provide an effective treatment to preserve muscle volume [16]. Maddocks’ work in cancer patients [13, 19] however did not demonstrate a significant increase in muscle volume and therefore one may question the rationale behind use in this patient group (these differences are summarised in Table 1). The cancer population in these studies is different from our own in a number of respects beyond the diagnosis alone and as such we may find NMES to be a more suitable intervention in our patient group. Maddocks’ work was performed in a palliative population with active cancer whilst postoperatively our patients will be theoretically cancer free with perhaps a few exceptions in patients who have solitary metastases (which, by the criteria of inclusion, are amenable to curative treatment). In view of their palliative status, Maddocks’ population would be expected to decline in health over time whilst our population would be expected to make a recovery up to or even beyond their preoperative state and therefore NMES may increase the rate of or facilitate this recovery. Our population is confined to bed rest for over a week’s duration following surgery and therefore activity provided by NMES may help arrest the muscle loss associated with disuse as in Hasegawa’s population whose anterior cruciate ligament repair cohort was subject to muscle loss through disuse rather than disease [16]. Finally, our patients will receive intensive inpatient support by the ward physiotherapists and the research team; they will receive positive reinforcement of their use of the device and will be asked to complete an exercise diary which the physiotherapy team, will review with them at each point they receive formal physiotherapy sessions. This level of direct input and positive reinforcement is notably more than in the previous NMES studies of Maddocks’ and therefore we would hope compliance and correct usage would be increased.

The inflammatory effects of exercise are known to be paradoxical in that exercise drives both a pro and anti-inflammatory response [32, 33]. We propose that the metabolic result of exercise in cancer patients will drive a beneficial anti-inflammatory response. This immunomodulation may in part help support the body’s immune system in the early stages of post-surgical recovery and as such may potentially support the cellular immune system in being able to identify and destroy malignant cells shed at the time of surgery.

In our patient group, NMES will potentially allow a higher degree of exercise than the patients would otherwise be able to undertake due to their incapacity. Our hope is that this promotes muscle preservation, allowing earlier mobilisation and a more expedient return to “normal” exercise and function, further reinforcing the preservation of muscle mass. Increased muscle mass and quality are associated with improved long-term outcome such as disease-free survival [6] we intend to follow our cohort for 5 years to see if NMES provides evidence to support improvement in these oncological outcomes through muscle preservation.

## Summary

Exercise in healthy individuals leads to increased muscle mass; exercise can bring about an anti-inflammatory effect due to muscle physiology thus obfuscating a key pathway driving secondary sarcopenia. Preservation of muscle mass through early post-operative intervention with NMES would allow a more rapid return to normal exercise and normal function leading to greater muscle preservation and subsequently improved outcomes.

### Trial status

BiCyCLE NMES is currently recruiting patients; recruitment began on the 31 May 2019 and is expected to be completed by March 2021. Protocol version 6 dated 05/06/20 is currently approved by the research ethics committee and the HRA.

## Supplementary Information

**Additional file 1.**

**Additional file 2.**

**Additional file 3.**

## Data Availability

Access to the final trial data set will be available to the trial team, the sponsor and for review by the independent data monitoring committee appointed by the sponsor.
