# Supplementary material for: BiCyCLE NMES—neuromuscular electrical stimulation in the perioperative treatment of sarcopenia and myosteatosis in advanced rectal cancer patients: design and methodology of a phase II randomised controlled trial
Source: Trials. 2021 Sep 15;22:621. doi: 10.1186/s13063-021-05573-2 (PMC8442432; doi:10.1186/s13063-021-05573-2)
Supplement: Supplementary file 1 — Additional file 1. [file 13063_2021_5573_MOESM1_ESM.pdf]

NHS Health Research Authority

Putting patients at the HEART of everything we do

St MARK'S ACADEMIC INSTITUTE

NHS London North West University Healthcare NHS Trust

BiCyCLE NMES – Electric Bike

BiCyCLE

Body Composition manipulation in CoLorectal cancEr

Protocol V6.0 05/06/20 IRAS: 242002

BiCyCLE NMES – Electric Bike

Body Composition Manipulation in Colorectal Cancer (BiCyCLE): Neuro-Muscular Electrical Stimulation (NMES) and its effect on the systemic inflammatory response and changes in body composition following radical surgery for locally advanced rectal cancer – a single centre double blind randomised controlled phase II clinical trial

Full title of the study

Body Composition manipulation in CoLorectal cancEr (BiCyCLE): Neuro-Muscular Electrical Stimulation (NMES) and its effect on the systemic inflammatory response and changes in body composition following radical surgery for locally advanced rectal cancer – a single centre double blind randomised controlled phase II clinical trial

Short study title / acronym

BiCyCLE NMES – Electric Bike

Protocol version number and date

Version 6.0 05.06.20

Research reference numbers

IRAS Number: 242002

SPONSORS Number: RD18/115

FUNDERS Number: N/A

REC Reference: 19/LO/0259

Signature page

The undersigned confirm that the following protocol has been agreed and accepted and that the Chief Investigator agrees to conduct the study in compliance with the approved protocol and will adhere to the principles outlined in the Declaration of Helsinki, the Sponsor's SOPs, and other regulatory requirement.

I agree to ensure that the confidential information contained in this document will not be used for any other purpose other than the evaluation or conduct of the investigation without the prior written consent of the Sponsor

I also confirm that I will make the findings of the study publicly available through publication or other dissemination tools without any unnecessary delay and that an honest accurate and transparent account of the study will be given; and that any discrepancies from the study as planned in this protocol will be explained.

For and on behalf of the Study Sponsor:

Signature: .... Date: ....../....../......

Name (please print):
Mr Simon Lewis
Position: Head of Research Operations & Governance

Chief Investigator:

Signature: .... Date: ....../....../......

Name: (please print):
Mr John T Jenkins

List of contents

Full title of the study iii

Short study title / acronym iii

Protocol version number and date iii

Research reference numbers iii

Signature page iv

List of contents v

Key study contacts viii

Study summary ix
- Funding and support in kind x
- Role of study sponsor and funder x
- Protocol contributors xi
- KEY WORDS: xii
- STUDY FLOW CHART (Figure 1) xiii

Background 1

Rationale 3

Theoretical framework 7
- Introduction and patient selection 7
- Preoperative workup 8
- The treatment NMES arm: 9
- The placebo NMES arm: 9
- Follow up 9

BiCyCLE NMES – Electric Bike

Research question 10

Objectives 10

Outcomes 11
Primary outcome: 11
Secondary Outcomes: 11

Study design, methods of data collection and data analysis 12
Introduction and patient selection 12
Randomisation 12
Preoperative workup 12
The treatment arm: 13
The placebo NMES arm: 14
Follow up 15
Following Treatment 16
Data analysis 16
Dissemination of findings and results 17
Data management 17

Study setting 17

Sample size and recruitment 18
Eligibility Criteria 18
Inclusion criteria 18
Exclusion criteria 19
SAMPLE SIZE 19
STATISTICAL ASSESMENT OF SAMPLE SIZE 19
Sampling technique 21
Recruitment 21
Sample identification 22
Consent 22
Withdrawal Criteria 22

Ethical and regulatory considerations 23
Assessment and management of risk 23

Research Ethics Committee (REC) and other Regulatory review & report 24

BiCyCLE NMES – Electric Bike

Regulatory Review & Compliance 24
Amendments 24
Peer review 25
Patient & Public Involvement 25
Protocol compliance 26
Data protection and patient confidentiality 26
Indemnity 26
Access to the final study dataset 26

Study management 27

Trial Data Monitoring Committee 27

Dissemination policy 27
Dissemination policy 27
Authorship eligibility guidelines and any intended use of professional writers 27
Publication Policy 28
REFERENCES 28

Appendices 29
Appendix 1- a summative list of submitted documentation 29
Appendix 2 – Study flowchart with typical patient time points 30
Appendix 3 – Amendment History i
Appendix 4 – Data capture fields ii

Demographics ii
Appendix 5 – Patient group comments and criticisms (PPI representatives BG, LG, LJ, TW & RM) iii
Appendix 6 – Contraindications and cautions of NMES v

vi
Appendix 7 – Schedule of Events 1

Key study contacts

Chief Investigator	Mr J T Jenkins i.jenkins@nhs.net 020 8235 4177<br>St Mark's Hospital, London North West University Healthcare NHS Trust, Watford Road, Harrow, HA1 3UJ
Principle Investigator and Study Co-ordinator	Mr E T Pring edward.pring@nhs.net 020 8235 4177<br>St Mark's Hospital, London North West University Healthcare NHS Trust, Watford Road, Harrow, HA1 3UJ
Sponsor	Mr Simon Lewis simon.lewis4@nhs.net 0208 869 2011<br>Head of Research Operations & Governance, London North West University Healthcare NHS Trust<br>Northwick Park Hospital, Watford Road, Harrow, HA1 3UJ
Funder	St Mark's Hospital Foundation (Registered Charity number 1140930) St Mark's Hospital, Watford Road, Harrow, HA1 3UJ
Key Protocol Contributors	Mr E T Pring edward.pring@nhs.net 020 8235 4177<br>Clinical Research Fellow & Speciality Trainee in Surgery<br>St Mark's Hospital, London North West University Healthcare NHS Trust, Watford Road, Harrow, HA1 3UJ<br>Mr J T Jenkins i.jenkins@nhs.net 020 8235 4177<br>Consultant Colorectal Cancer Surgeon<br>St Mark's Hospital, London North West University Healthcare NHS Trust, Watford Road, Harrow, HA1 3UJ<br>Dr P Lung philliplung@nhs.net<br>Consultant Radiologist<br>St Mark's Hospital, London North West University Healthcare NHS Trust, Watford Road, Harrow, HA1 3UJ<br>Dr G Malietzis g.malietzis@imperial.ac.uk 020 8235 4177<br>Clinical Lecturer & Speciality Trainee in General Surgery<br>St Mark's Hospital, London North West University Healthcare NHS Trust, Watford Road, Harrow, HA1 3UJ<br>Prof J Saxton john.saxton@northumbria.ac.uk 01912 273 371

	Head, Department of Sport, Exercise and Rehabilitation, Faculty of Health and Life Sciences, Northumbria University, Newcastle Upon Tyne, NE18ST<br>Dr C Taylor clairetaylor8@nhs.net 020 8869 2472<br>Nurse Consultant Complex Colorectal Cancer<br>St Mark's Hospital, London North West University Healthcare NHS Trust, Watford Road, Harrow, HA1 3UJ<br>Dr M Naghibi maninaghibi@nhs.net 020 8235 4177<br>Consultant in Gastroenterology and Nutrition<br>St Mark's Hospital, London North West University Healthcare NHS Trust, Watford Road, Harrow, HA1 3UJ<br>Miss D Chauhan deeptika.chauhan@nhs.net 020 8869 2228<br>Specialist Physiotherapist<br>Northwick Park Hospital, London North West University Healthcare NHS Trust, Watford Road, Harrow, HA1 3UJ<br>Miss L Gould laura.gould3@nhs.net 020 8235 4177<br>Research Fellow Colorectal Surgery and Nutrition<br>St Mark's Hospital, London North West University Healthcare NHS Trust, Watford Road, Harrow, HA1 3UJ
Medical Statistician	Mr P Bassett paul@statsconsultancy.co.uk<br>Medical Statistician<br>Northwick Park Institute of Medical Research (NPIMR), Northwick Park Hospital, Watford Road, Harrow, HA1 3UJ

Study summary

Study Title	Body Composition Manipulation in Colorectal Cancer (BiCyCLE): Neuro-Muscular Electrical Stimulation (NMES) and its effect on the systemic inflammatory response and changes in body composition following radical surgery for locally advanced rectal cancer – a single centre double blind randomised controlled phase II clinical trial
Internal ref. no. (or short title)	BiCyCLE NMES – Electric Bike
Study Design	A single centre double blind randomised control trial

BiCyCLE NMES – Electric Bike

Study Participants	Patient with primary or recurrent locally advanced rectal cancer who are undergoing radical/curative pelvic surgery
Planned Size of Sample (if applicable)	58 patients – 29 in treatment arm and 29 in the placebo group
Follow up duration (if applicable)	5 years from the date of surgery or until death of patient or patient withdrawal from trial or loss of contact with patient
Planned Study Period	Recruitment and treatment 2 years. Routine data collection for 7 years from start of study. Recruitment opens March 2019 closes March 2021. Final trial specific measurements March 2022. Long term follow up concludes and end of study March 2026.
Research Question/Aim(s)	Does pre and post-operative neuro muscular electrical stimulation decrease myosteatosis, increase muscle mass, decrease the systemic inflammatory response and improve short and long term outcomes in patients undergoing radical surgery for primary or recurrent locally advanced rectal cancer?

Funding and support in kind

**FUNDERS**	**FINANCIAL AND NON FINANCIALSUPPORT GIVEN**
St Mark's Hospital Foundation (Registered Charity number 1140930) St Mark's Hospital, Watford Road, Harrow, HA1 3UJ	Funding for the clinical research fellow salary, funding for consumables and stationary, funding for the MicroStim 2v2 stimulators and their disposable attachments. Training of physiotherapist and nursing staff to use the device.
London North West University Healthcare NHS Trust	The Sponsor will cover all costs of routine clinical care i.e. surgery, hospital stay, routine blood tests, radiological imaging and post operative follow up.

Role of study sponsor and funder

London North West University Healthcare NHS Trust (LNWUH) is the trial sponsor and has delegated the overall management, including study design, conduct, data analysis interpretation and dissemination of results, of the BiCyCLE NMES trial to the CI and PI/Study Coordinator. The trial will take place at St Mark's Hospital, part of LNWUH and consequently patients from within the trust will be recruited into the trial. The R&D department of LNWUH will

be involved in assisting the administration of the trial design and will be responsible for monitoring the trial and auditing it.

Neither the Sponsor nor Funder will be involved in the decision of which findings will be included within the final work.

Protocol contributors

The trial sponsor and funder has delegated the overall management, including study design, conduct, data analysis interpretation and dissemination of results, of the BiCyCLE NMES trail to the CI and PI/Study Coordinator. The trial will take place at St Mark's Hospital, part of LNWUH NHS Trust and thusly patients from within the trust will be recruited into the trial.

The protocol has been designed by the BiCyCLE Research Study Group involving the CI and PI/Study Coordinator. The Sponsor and funder will not be involved in the protocol design. The R&D department of LNWUH will review the study protocol and be involved in the audit and monitoring of the trial. The Sponsor will not be involved in scientific construction of the study. The funder will not be involved in the study design nor in the decision of which data is included within the write up of results nor the dissemination of scientific material within the study. LNWUH NHS Trust has delegated the final decision of what to publish to the CI.

BiCyCLE NMES – Electric Bike

KEY WORDS:	NMES, neuro-muscular electrical stimulation, sarcopenia, myopenia, rectal cancer, systemic inflammatory response, double blind randomised control trial, Microstim 2v2

BiCyCLE NMES – Electric Bike

STUDY FLOW CHART (Figure 1)

Inclusion Criteria:

- Adults age 18 to 99
- Male or female
- Primary or Recurrent locally advanced rectal cancer amenable to elective radical surgery
- ASA I-III
- Able and willing to consent
- Participation in other concurrent trials acceptable – following discussion with trial team.

Exclusion Criteria

- Lack of patient consent
- Contraindication to NMES
- Pre existing neuromuscular degenerative disease
- Participation in other trials where agreement on participation not made in advance by trial teams

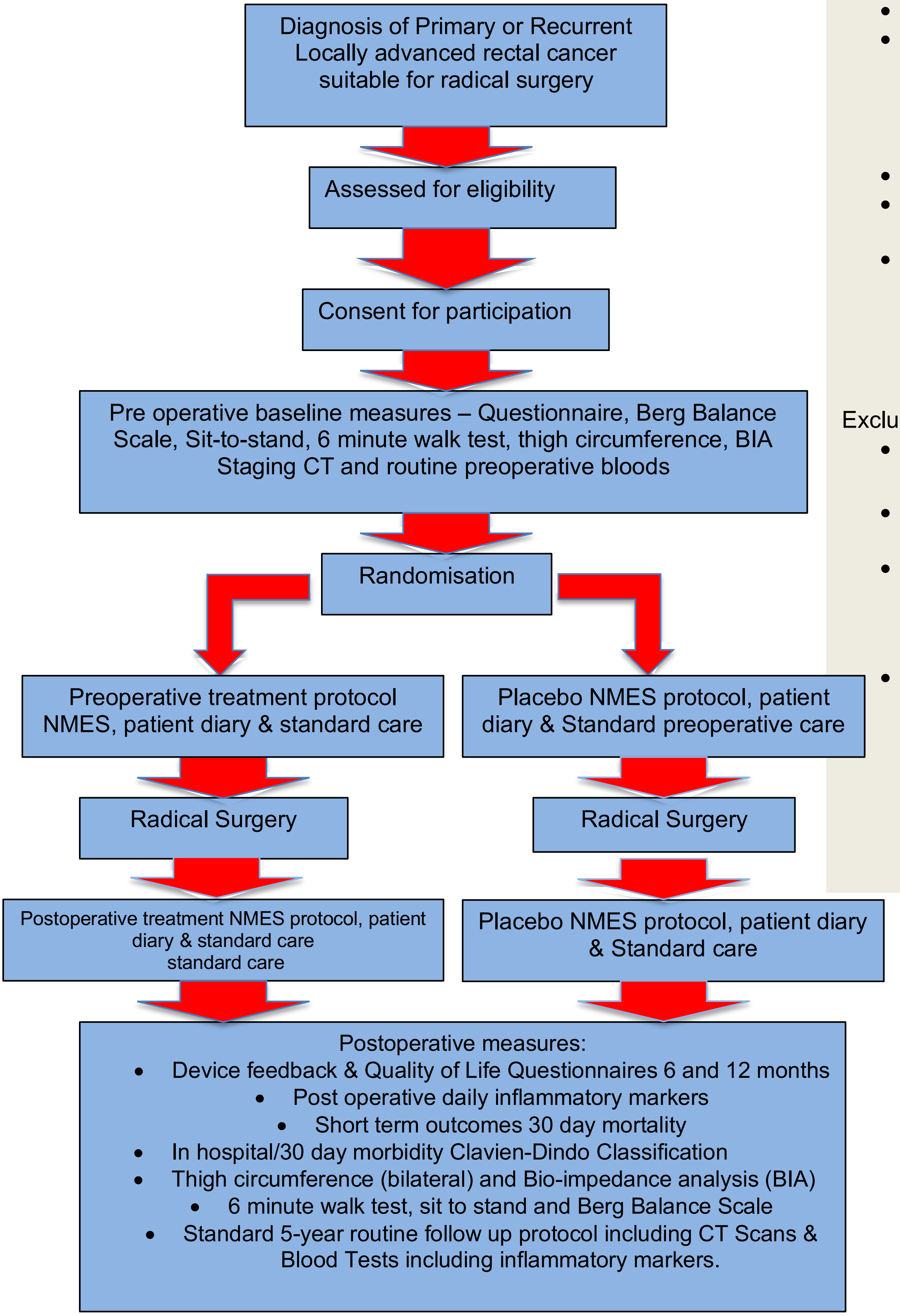

BiCyCLE NMES – Electric Bike

Study protocol

Body Composition Manipulation in Colorectal Cancer (BiCyCLE): Neuro-Muscular Electrical Stimulation (NMES) and its effect on the systemic inflammatory response and changes in body composition following radical surgery for locally advanced rectal cancer – a single centre double blind randomised controlled phase II clinical trial.

Background

As part of the syndrome and disease process of cancer the body undergoes changes in both its muscle and fat collectively known as body composition. This alteration occurs for a number of reasons including inflammatory changes, hormonal changes, loss of function, fatigue and energy balance. These factors are compounded following major gut surgery. Loss of muscle mass and quality, known as sarcopenia/myopenia (figure 2), has been shown to be associated with poorer postoperative outcomes following surgery for colorectal cancer(1,2). If we can preserve muscle mass, quality and function we may be able to improve these outcomes.

Exercise, by repetitive muscle use, physiologically leads to an increase in muscle mass. Exercise also imparts an anti-inflammatory effect by attenuating the cellular response to inflammatory stimuli and pro-inflammatory cytokines (3) (4). By virtue of these two statements one can suggest that prescribed exercise could be a powerful tool in the treatment, management and even prevention of muscle wasting in cancer. However, exercise following major surgery is not always possible or practical and therefore alternatives have been sought. One such example of major surgery is pelvic exenteration – major pelvic surgery to excise all organs within the pelvis, which are directly involved by the invasive cancer.

One such alternative could be neuromuscular electrical stimulation (NMES). The use of exogenous electrical stimulation of muscle has the potential to directly increase muscle mass by mimicking the process of exercise. A battery powered stimulatory unit is secured by self-adhesive electrodes to the patient's skin superficial to the muscle body and once activated produced smooth regular contractions. A typical program consists of 30 to 60 minutes of stimulation, generally of the quadriceps with or without additional lower limb muscles, for example calves, hamstrings, or glutei, three to five times each week, for four to eight weeks (5). NMES can be used to produce a muscle contraction equivalent to 20% to 40% of a maximum

voluntary contraction (6) thus meeting the criteria of the American College of Sports medicine definition of planned exercise (7). It is. Important to note that NMES is not a perfect surrogate for "normal" exercise as it is recognised that the rate of fatigue during the therapeutic application of electrical stimulation of skeletal muscle is much greater than that seen during voluntary contractions(8). NMES will however allow a higher degree of exercise than the participants would otherwise be able to undertake due to their incapacity. Our hope is that this promotes muscle preservation, allowing earlier mobilisation and a more expedient return to "normal" exercise and function, further reinforcing the preservation of muscle mass.

Functional electrical stimulation (FES) using NMES is used in clinical practice for a number of diseases, indeed, at the National Clinical FES Centre at Salisbury NHS Foundation Trust over 2500 patients are currently undergoing FES(9). NMES has been shown to increase muscle bulk in a healthy limb. In anterior cruciate ligament [of the knee] (ACL) reconstruction patients, NMES implemented during the early rehabilitation stage is effective in maintaining and increasing muscle thickness and strength in the operated limb (10). There is also evidence from meta-analyses that NMES increases muscle strength and shows potential benefit for joint range of motion, muscle atrophy, outcomes of ventilation and activity limitations in critically ill patients(11). A Cochrane review of NMES in a number of diseases which cause cachexia (muscle and fat loss secondary to disease) such as COPD (Chronic obstructive pulmonary disease), CCF (Congestive cardiac failure), HIV/AIDS and cancer suggested NMES may be an effective treatment for muscle weakness in adults with advanced progressive disease, and could be considered as an exercise treatment for use within rehabilitation programs (5).

Two of the studies, a phase 2 randomised trial and its pilot study, included in this review looked specifically at cancer cachexia. Both studies were conducted in patients with non-small cell lung cancer receiving palliative chemotherapy. The pilot study demonstrated positive results (12) however, the phase 2 study of 49 patients in which 30 were randomized to NMES it was found that there were no significant differences in quadriceps muscle strength, thigh lean mass or physical activity level between groups (13). The study team did however recommend further study in NMES in patients with cancer in other settings.

Our study as well as describing changes in body composition between both groups would also be outcome related – post surgical complications, quality of life, function, disease free

survival (DSF) and overall survival (OS). The study would also examine the relationship between muscle mass, exercise and the systemic inflammatory response, which through previous work has been shown to correlate with outcomes. Our study population and design differ markedly from Maddocks' and these differences are summarised below and described in detail within our study rationale. It is for these reasons that we feel that NMES could be a potent treatment in this group.

Our Study Population	Maddock's Study Population
**Post-operative "tumour free"**	Active cancer
**Confined to bed rest**	Active and mobile population
**Intensive inpatient support**	Outpatient community care
**Aiming for recovery up to or beyond baseline**	Palliative and functionally declining population

Rationale

Study Aim: To ascertain whether pre and post operative NMES of the quadriceps and core muscles of the lower back decreases myosteatosis, increases muscle mass, decreases systemic inflammation and improves short and long term outcomes following radical pelvic surgery for locally advanced rectal cancer.

As described above there is proposed association between muscle mass and outcomes in cancer. Evidence suggests that decreased muscle mass (sarcopenia), which is multifactorial in nature (see figure 2), during the development of cancer leads to poorer outcomes both in the immediate postoperative period but also in long term outcomes and prognosis. Our belief is that exercise through multiple physiological pathways may preserve muscle mass by virtue of the following statements:

- Exercise in healthy individuals leads to increased muscle mass
- The process of exercise brings about an anti-inflammatory effect due to muscle physiology thus decreasing myopenia secondary to the inflammatory process of cancer
- Preservation of muscle mass through early intervention would allow a more rapid return to normal exercise and normal function

BiCyCLE NMES – Electric Bike

Figure 2.

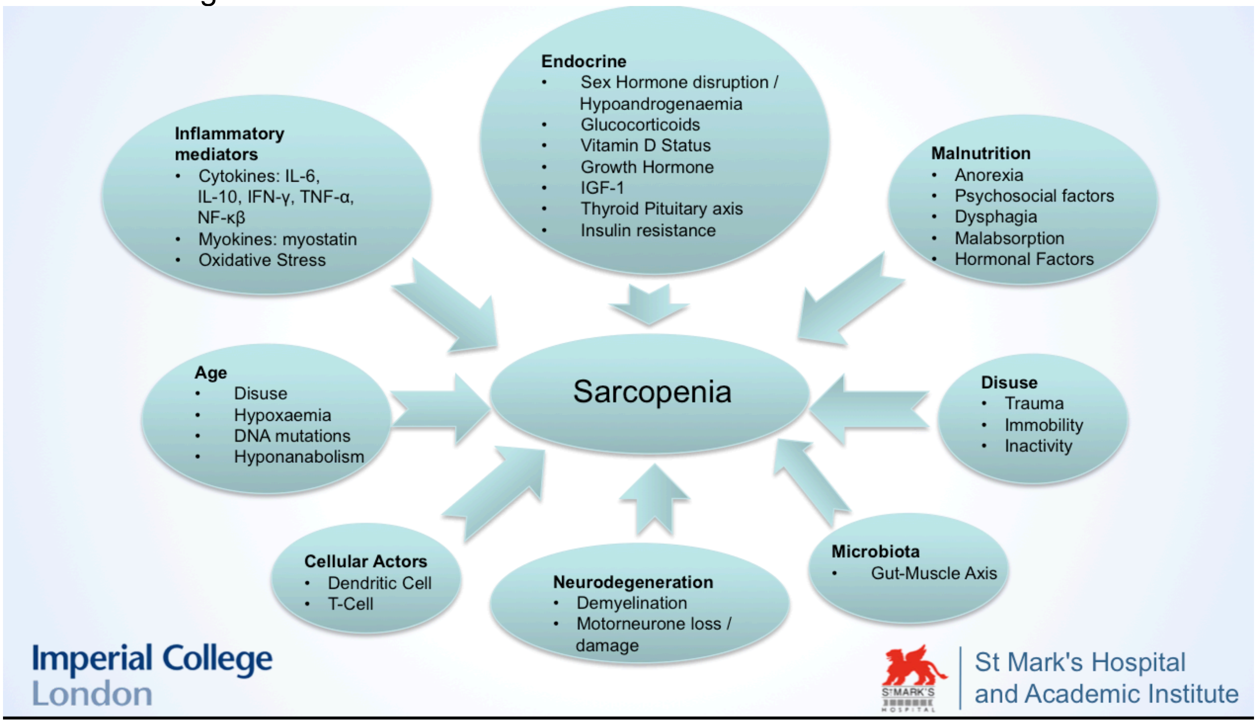

Following exenterative pelvic surgery patients find it difficult to mobilise, NMES would therefore be a suitable alternative to mobilisation as it can be conducted in the patient's own bed. NMES can be used to produce a muscle contraction equivalent to 20% to 40% of a maximum voluntary contraction (6) thus meeting the criteria of the American College of Sports medicine definition of planned exercise (7). A Cochrane review of NMES in a number of diseases which cause cachexia such as COPD, CCF, HIV/AIDS and cancer suggested NMES may be an effective treatment for muscle weakness in adults with advanced progressive disease, and could be considered as an exercise treatment for use within rehabilitation programs (5). NMES has been shown to increase muscle bulk in a healthy limb for example in ACL reconstruction patients NMES implemented during the early rehabilitation stage is effective in maintaining and increasing muscle thickness and strength in the operated limb (10).

We have seen from this study by Hasegawa (9) that muscle tissue can be maintained or increased in disuse following ACL reconstruction. By virtue of this we know that in muscle disuse NMES may provide an effective treatment to preserve muscle volume. Maddocks' work in cancer patients however did not demonstrate a significant increase in muscle volume and therefore one may question the rationale behind use in this patient group. The cancer population in these studies is different from our own in a number of respects beyond the

diagnosis alone and as such we may find NMES to be a more suitable intervention in our patient group. Maddocks' work was performed in a palliative population with active cancer whilst postoperatively our patients will theoretically be cancer free with perhaps a few exceptions in patients who have solitary metastases (which, by the criteria of inclusion, are amenable to curative treatment). In view of their palliative status Maddocks' population would be expected to decline in health over time whilst our population would be expected to make a recovery up to or even beyond their preoperative state and therefore NMES may increase the rate or facilitate this recovery. Our population is confined to bed rest for over a week's duration following surgery and therefore activity provided by NMES may help arrest the muscle loss associated with disuse as in Hasegawa's population. Finally, our patients will receive intensive inpatient support by the ward physiotherapists and the research team at St Mark's, they will receive positive reinforcement of their use of the device and will be asked to complete an exercise diary which the physiotherapy team, will review with them at each point they receive formal physiotherapy sessions. This level of direct input and positive reinforcement is notably more than in the previous NMES studies of Maddocks' and therefore we would hope compliance and correct usage would be increased.

No work has been done to date on NMES in colorectal cancer and of the other studies performed in cancer patients there has been no examination of the relationship to the systemic inflammatory response nor has there ever been an assessment of immediate post-operative outcomes. Our proposed method of examining muscle mass and quality on CT (SliceOmatic software) has also been validated and could be easily translated into regular clinical practice potentially introducing the assessment of Sarcopenia into regular clinical prognostication.

We will endeavour to stimulate two major muscle groups during the study. The muscles of the thigh particularly vastus lateralis and vastus medialis will be stimulated in both legs, this will be performed with a view to preserving muscle mass and encouraging earlier ambulation and better function. We will also stimulate the erector spinae muscles, and the muscles of the lower back. The reason for this is twofold, firstly it is felt that some of the earliest muscles to atrophy following surgery or during bed rest are the core muscles of the back especially as these patients will not be sitting up or utilising these important supportive muscles in the first stages of their recovery. Patients are nursed on their side during the first 10 days following major pelvic surgery, which means they tend not to use their core muscles to flex or extend their back or

support their weight leading to loss. This lateral position however would afford easy access to place the electrodes. Secondly, we are focussing on the muscle groups at the L3 level – the level this stimulation would take place, using the device in this location would give us the best chance of demonstrating the benefits of the device with regards muscle preservation. This site is well away from the operative site and tumour bed in these individuals and therefore there would be no risk of stimulating the tumour bed whilst using the device in this position.

We recognise that stimulation of the lower back will be technically challenging at home or during independent use we therefore will only perform lower back stimulation during admission whilst the patient is immobilised. Once the patient is mobile and supporting their torso independently we would suppose that there would be little further benefit from continued lower back stimulation.

It is important that we measure not only the anatomical effects of NMES i.e. increased muscle mass on CT and anthropometric changes but we identify whether these patients demonstrate both a functional and physiological improvement. To that end we will assess functionality preoperatively at diagnosis and post operatively at 3 months using the validated instruments of the 6-minute walk test, Sit-to-stand and Berg Balance scale. We will measure thigh circumference at 15cm above the superior pole of the patella (which has been shown in earlier studies to correlate with muscle volume on MRI)(14). We will also examine quality of life and patient experience of using the device. Patient physiology will be assessed by short- and long-term operative outcomes and assessment of postoperative inflammatory profile. Due to the advanced disease and radical nature of the surgery we will not examine bladder and bowel function as many of these patients have cystectomies (resection of the bladder) and proctectomies (resection of the rectum) and subsequent permanent urostomies and end ileostomies or colostomies.

The inflammatory effects of exercise are known to be paradoxical in that exercise drives both a pro and anti-inflammatory response. We propose that the metabolic result of exercise in sarcopenia will drive a beneficial anti-inflammatory response. This immunomodulation may in part help support the body’s immune system in the early stages of post-surgical recovery and as such may potentially support the cellular immune system in being able to identify and destroy malignant cells shed at the time of surgery. To monitor the inflammatory response, we will use commonly utilised postoperative inflammatory markers, namely CRP and values derived from

the full blood count and biochemistry including NLR and mGPS. These inflammatory markers are well-established metrics linked to prognosis in colorectal cancer. We have chosen these markers for a number of reasons; they are routinely taken, cost effective and allow for comparison with substantial historical data. We may also require results from other trusts, due to the national spread of our patient population, and we cannot support them in obtaining non-routine tests as part of this study. More esoteric markers such as the putative inflammatory mediator IL6, which has been implicated in the inflammatory process of sarcopenia, have not been chosen to be measured in this study. Although tests for IL6 are available they are expensive and the body of evidence for their role in post-surgical recovery is somewhat limited. There is some suggestion in the literature that FES also has the potential to aid wound healing, however due to the complex nature and positioning of wounds in these patients we will not be examining this as part of this study, however wound complication data is kept routinely and will form part of our analysis but wounds will be away from the sites of direct stimulation.
Taking into account all the above statements we devised this project to address these unknowns and drive forward the multifaceted and holistic approach to cancer therapy, pre and rehabilitation.

Theoretical framework

Introduction and patient selection

This study is designed to meet and will be performed in line with the CONSORT criteria (http://www.consort-statement.org/consort-2010). Following diagnosis of locally advanced rectal cancer patients are discussed in a multidisciplinary team (MDT) meeting. Some of these patients may be felt to be suitable for radical surgery – i.e. surgery performed with the intention of a cure. These patients are reviewed and discussed in a tertiary centre such as St Mark's Hospital (LNWUH NHS Trust). If patients are deemed fit for and consent to surgery then this is performed by one of two specialist surgeons in St Mark's Hospital.

Patients who undergo major pelvic surgery have limited mobility due to postoperative pain and disability. These patients are therefore at much greater risk of suffering from muscle wasting than

patients undergoing more routine colorectal surgery. This is a result of greater loss of function, greater immobility and potentially a more profound immunogenic inflammatory response.

Currently these patients receive postoperative physiotherapy, due to limited time, postoperative pain, patient choice and resource availability it is unlikely that the patients are exercised to their full potential. A prescribed program with a NMES device would allow patients to choose when they undertake muscle stimulation exercise for example once they had received adequate analgesia or at a time convenient to them. This would hopefully improve compliance and bring about a hypertrophic response in the muscle.

We would therefore compare NMES and current best practice against current best practice alone.

NMES would be used preoperatively to get patients used to and increase confidence in using the device prior to surgery but also to intervene as soon as possible in muscle recruitment and preservation as earlier intervention is thought to preserve more muscle and function, this process is called pre-habilitation. This is especially important in a cancer population as the pathological process of cachexia and deleterious changes in body composition occur as part of the cancer process often preceding any medical or surgical intervention. These changes in body composition are an important focus on our work and it's through this pre-habilitation phase, by the earliest possible intervention that we would hope to help arrest these changes.

The intervention period of the study will last 2 years by which time we hope to have recruited sufficient numbers, by the end of a 3-year period all patients should have completed their quality of life questionnaires. After this period the remaining duration of the trial will involve data collection from the routine hospital visits of the patient population taking the total duration of the study to 7 years.

A summary of the intervention is described below with a fuller and more detailed explanation in the next section.

Preoperative workup

As part of their preoperative work up for surgery patients would have blood tests including inflammatory markers, a staging CT scan of their chest abdomen and pelvis and occasionally CPET (cardiopulmonary exercise testing). Patients who meet the inclusion criteria will be identified by the clinical team and will then be approached by the study team, either the research fellow or

colorectal study coordinator, and given the option to enrol in the study following which consent will be obtained.

The treatment NMES arm:

Patients will be blinded as to which arm of the trial they are in. Neuromuscular stimulation will be delivered by a MicroStim Exercise Stimulator MS2v2 (Odstock Medical Limited, Wiltshire, UK) using two self-adhesive electrodes placed on the anterior thigh over the body of the vastus medialis and vastus lateralis muscles and the lower back.

Patients in the treatment arm will also receive standard care including nutritional support and physiotherapy in line with current guidelines and local hospital practices. Routine daily blood tests for inflammatory markers will be taken until discharge, as is current practice.

The placebo NMES arm:

The same model of MicroStim Exercise Stimulator MS2v2 (Odstock Medical Limited, Wiltshire, UK) will be provided to the placebo group who will apply two self-adhesive electrodes placed on the anterior thigh over the body of the vastus medialis and vastus lateralis muscles and the lower back as in the treatment group. This will be programmed to provide sub-therapeutic electrical stimulation.

Patients will receive standard care including physiotherapy and nutritional support in line with current hospital guidelines and practices. Routine daily blood tests for inflammatory markers will be taken until discharge, as is current practice.

Follow up

Patients from both the treatment and control arms will receive standard five year follow up. Histopathological data will be recorded following processing of the resected specimens by the pathologist. Quality of Life data, patient satisfaction, Bio-impedance analysis, CT Body composition and functional measurements will be taken as detailed below.

Early stage follow up to identify changes within the pre and post-operative CT scans and analysis of NMES satisfaction and the initial inflammatory data will take place at 3 months following the recruitment of the final patient. We will then continue long term follow up for the standard 5 year

follow up period or until patient death. Final analysis will take place at 5 years following recruitment of the final patient.

Research question

To evaluate the effect of pre and post-operative NMES on muscle quality and lean body mass; postoperative systemic inflammation; postoperative complications, quality of life and recovery in patients undergoing exenterative surgery for locally advanced rectal cancer.

Objectives

In comparison to our control group receiving standard care we aim to:

- To assess the effect of NMES on body composition, muscle surface area and muscle quality at the L3 vertebral level on the routine pre and serial post-operative CT scans following radical pelvic surgery.
- To assess the effect of NMES on systemic inflammation following radical pelvic surgery for locally advanced rectal cancer.
- To assess the effect of NMES on short-term outcomes following radical pelvic surgery.
- To assess the effect of NMES on long term outcomes including disease free survival (DFS) and overall survival (OS) following radical surgery for locally advanced rectal cancer.
- To assess the effect of NMES on quality of life following radical surgery for locally advanced rectal cancer.
- To ascertain if the NMES is well tolerated and easy to use in the post surgery setting and to assess patient compliance with the prescribed treatment.

Outcomes

Primary outcome:

The difference in mean muscle attenuation (MA) measured in Hounsfield units between the pre-operative and 3 month post-operative CT scan in the NMES treatment group and the placebo NMES group.

Secondary Outcomes:

- The difference in LSMI and VAT derived from the L3 vertebral axial level of pre and 3 to 6 month postoperative CT scans using SliceOmatic software
- Pre and Post-operative inflammatory markers (CRP, GPS and NLR)
- Short-term outcomes including complications, defined by the Clavien-Dindo classification for postoperative complications.
- Length of hospital stay
- Disease Free Survival
- Overall survival
- Post-operative quality of life at 6 months and 12 months following surgery using the validated quality of life questionnaire ED-5Q-5L & EORTC QLQ – CR29
- Assessment of compliance and tolerability of the NMES device in the post-surgical setting
- Function as assessed by the Berg Balance scale, sit-to-stand and 6-minute walk test at baseline and approximately 3 months following surgery.
- Difference in thigh circumference both legs (at 15cm above the superior pole of the patella) preoperatively and at 3 month postoperatively.
- Sequential changes measure on CT over a 5-year follow-up period following surgery.
- Dose response to NMES utilising the patient compliance diary and data from the device activity reader and CT derived data.
- CPET data and response to NMES as demonstrated by changes in body composition.
- Patient satisfaction with using the NMES device.
- Bio-impedance analysis (BIA) metrics at set time points (baseline, day two post operatively, day twenty-eight post operatively (if in hospital) day of discharge, first post-operative follow u appointment.

Study design, methods of data collection and data analysis

Appendix 2 shows the study flow chart with ideal patient intervention time points.

Introduction and patient selection

The BiCyCLE NMES Trial is a single centre double blind randomised controlled trial, patients will be blinded as to which trial arm they enter and a sham protocol will be used by the control arm. Body composition analysis of the images will be done by automated software used by an operator blinded to the intervention to remove operator or interpretation bias.

Following diagnosis of locally advanced rectal cancer patients are discussed in a multidisciplinary team (MDT) meeting. Some of these patients may be felt to be suitable for radical surgery – i.e. surgery performed with the intention of a cure. If patients are deemed fit for and consent to surgery then this is performed by one of two specialist surgeons in St Mark's Hospital.

Patients who meet the inclusion criteria will be identified by the clinical team in the colorectal outpatient clinic or MDT and will then be approached by the study team with written information on the trial and given the option to enrol in the study. Consent to take part in the trial will be obtained at the next outpatient clinic appointment, which will occur in the weeks preceding surgery.

Randomisation

Randomisation will take place by computer generated randomisation software (https://www.sealedenvelope.com) on a one to one basis. Patients who are randomised to the either arm will be blinded as to intervention and will be taught by the research team to use the stimulator this will be at their clinic appointment following consent.

Preoperative workup

As part of their preoperative work up for surgery patients have blood tests including inflammatory markers, a staging CT scan of their chest abdomen and pelvis and occasionally cardiopulmonary exercise testing (CPET). Anonymised data from these tests will be recorded from the clinical systems (ICE, SECTRA PACS) and patient notes into the study database by the

research team. The CT scans will be analysed by a blinded member of the research team using SliceOmatic v5.0 (TomoVision, Qc, Canada) with the ABACSL3 automated plug-in (Voronoi Health Analytics, BC, Canada) with the subsequent data recorded in the study database.

The treatment arm:

At their second clinic appointment at St Mark's patients will be trained by the research fellow or other competent research team member (physiotherapist or specialist nurse) to use the NMES. The NMES stimulator would be used preoperatively to get patients used to the device and increase confidence in using the device prior to surgery but also to intervene as soon as possible in muscle recruitment and preservation. This is important as the systemic disease of cancer itself leads to body composition deconditioning.

Neuro-muscular stimulation will be delivered by a MicroStim Exercise Stimulator MS2v2 (Odstock Medical Limited (OML), Wiltshire, UK) using two self-adhesive electrodes placed on the anterior thigh over the body of the vastus medialis and lateralis and the muscles of the lower back.

An study specific instruction leaflet will be given to this group along with the standard instruction manual by OML.

The program will commence preoperatively and consist of daily stimulation to one thigh at a time followed by the lower back each for 15 minutes, increasing to 60 minutes within one week as tolerated. One treatment session for both thighs would last between 60 to 90 minutes in total per day – this can be taken in up to three discrete sessions. Treatment will last for two weeks pre-operatively and at least four weeks postoperatively or until patient returns to independent ambulation.

Pulse waveform (symmetrical biphasic squared), frequency (40 Hz), and width (350 microseconds) would be used for the duration of treatment with the NMES. The amplitude (device output 0-120 mA, tested across 1000**Ω**) will be set to elicit a visible and comfortable muscle contraction; patients will be encouraged to subsequently increase the amplitude as tolerated. A "compliance diary" will be kept by the patients during their treatment period detailing their time spent using the device and the settings at which they are using it.

BiCyCLE NMES – Electric Bike

This program is adapted from one found to be of benefit in a pilot study of patients with non-small cell lung cancer which itself was based on an NMES exercise program developed for patients with COPD. The stimulation parameters were selected to favour gains in function and strength over endurance (frequency), minimise skin irritation (pulse width), and allow for sufficient recovery of the muscles between contractions (duty cycle)(12)(13).

Patients using the device will also complete a questionnaire on compliance, comfort and usability of the device in the postoperative setting. Qualitative data and free text comments from this may be used in the final written publications.

Patients in the treatment arm will also receive best standard care including parenteral nutritional support and physiotherapy in line with current guidelines and local hospital practices. Routine daily blood tests for inflammatory markers will be taken until discharge, as is current practice.

Data from blood tests and questionnaires completed during the inpatient stay will be anonymised and added to the database by the research team.

The placebo NMES arm:
At their second clinic appointment at St Mark's patients will be trained by the research fellow or other competent research team member (physiotherapist or specialist nurse) to use the NMES.

The patients will be blinded as to which trial arm they are in. The same model of MicroStim Exercise Stimulator MS2v2 (Odstock Medical Limited, Wiltshire, UK) will be provided to the placebo group who will apply two self-adhesive electrodes placed on the anterior thigh over the body of the vastus medialis and vastus lateralis muscles and the lower back as in the treatment group.

Patients will receive a study specific instruction leaflet with information on how to use the device in addition to the OML instruction manual. The program will commence preoperatively and consist of daily stimulation to one thigh at a time followed by the lower back each for 15 minutes, increasing to 30 minutes within one week. One treatment session for both thighs and lower back would last between 60 to 90 minutes in total per day – this can be taken in up to three discrete sessions.

Pulse waveform (symmetrical biphasic squared), frequency (40 Hz), and width (350

microseconds) would be used until the fourth postoperative week. The amplitude (device output 0-120 mA, tested across 1000**Ω**) will be set to produce an initial stimulation below motor threshold which will gradually reduce to 0mA over a ramp of approximately two minutes with a further ramp provided at the end of stimulation. This has been used as an effective placebo in transcranial neuro stimulation in a treatment naïve population. The stimulators light indicators will light up to mimic the device in the treatment group. Patients may experience a slight tingling sensation during the two-minute ramp period.

Patients using the device will also complete a questionnaire on compliance, comfort and usability of the device in the postoperative setting.

Patients will also receive best standard care including parenteral nutrition post operatively, physiotherapy and nutritional support in line with current hospital guidelines and practices. Routine daily blood tests for inflammatory markers will be taken until discharge, as is current practice.

Data from blood tests and questionnaires completed during the inpatient stay will be anonymised and added to the database by the research team.

Follow up

Surveillance CT scans are performed as standard in this patient group. Sequential screening (i.e. not emergency or non-routine imaging) CT image analysis using SliceOmatic version 4.3 software (TomoVision, Montreal, Quebec, Canada) will be performed. Total skeletal muscle and VAT surface area (cm2) will be evaluated on a single image at the third lumbar vertebra (L3) using HU thresholds of −29 to 150 for skeletal muscle, −50 to 150 for VAT and −190 to −30 for subcutaneous adipose tissues. CT body composition analysis of all the included images will be analysed using the ABACS L3 automated plug-in software, which complements SliceOmatic. The automated process will be directed by a radiologist/researcher who will be blinded to the treatment group of individual patients. The automated process provided by the ABACS L3 plug-in also removes the possibility of operator bias in the analysis of the images. The sum of skeletal cross-sectional muscle areas will be normalised for stature (m2) and reported as lumbar skeletal muscle index (LSMI) (cm2/m2). Routine bloods including CEA (Carcinoembryonic antigen) and inflammatory markers will be measured at each elective routine clinic visit and these data recorded. Quality of life will be assessed at 6 and 12 months

using validated quality of life questionnaires (ED-5Q-5L & EORTC QLQ – CR29). The Berg Balance scale, sit-to-stand test and 6 minute walk test will be used to assess functional outcome these tests will be performed at the patients 3 month post operatively clinic appointment. Pre and post-operatively we will measure bilateral thigh circumference at 15cm above the superior pole of the patella (which has been shown in earlier studies to correlate with muscle volume on MRI). Bio-impedance analysis (BIA) (a quick, non-invasive test for measuring body composition) will be undertaken at St Marks at set time points - baseline, day two post operatively, day twenty-eight post operatively (if in hospital) day of discharge and first post-operative follow up appointment. We will record data from the device satisfaction questionnaires from both groups. Standard outcome data will also be collected (appendix 4). Regarding patients whose treatment is followed up remotely, i.e. at different specialist units, we intend to collect relevant data via their local clinical nurse specialists at their home hospitals. We will request their CT scans be transferred, by the standard secure means, to St Mark's for analysis and follow up. We will obtain consent for this sharing of data from the patients.

Following Treatment

Following the completion of the ten-week treatment period within the trial patients will return the NMES device and no further NMES treatment will be given, the same is true of the placebo device.

Data analysis

Analysis will be based on intention to treat. Data from the CT scans will be analysed at the anatomical level of L3 using SliceOmatic Software v5.0 (TomoVision, Quebec, Canada), this analysis will be performed by the research team in our institution, this will provide anthropomorphic data on the participants' body composition in numerical form. Clinical outcome data will be coded and then processed. Numerical data collected as part of the study will be fully anonymised and analysed within St Mark's Hospital and Academic Institute. No patient identifiable data will leave the confines of the trust. Analysis will be performed using specialised statistical software (SPSS) and will be processed with the assistance of a clinical statistician in order to produce accurate and meaningful conclusions. Qualitative data may be used anonymously in the write up and presentation of the work to help elucidate and identify key ideas, which study participants, felt pertinent or relevant.

Dissemination of findings and results

The study findings will be published in peer-reviewed scientific journals, and presented at local, national and international meetings and conferences. Future researchers at St Mark's will be able to access a summary of information through the organisational website of St Mark's Hospital & Academic Institute. Data collected in this research database will not be shared with other individuals or research groups. Any future decision to share such data will require an application to and approval from an NHS Research Ethics Committee as part of a substantial amendment to study protocol.

Data management

The data collected will comply with the General Data Protection Regulations (GDPR) 2018. The day-to-day management of the data will be co-ordinated through the main research team members and overseen by the Principle Investigator Mr E T Pring. Data will be stored in a secure NHS protected computers at St Mark's Hospital. For further security and safety, data will be backed-up at regular intervals to a departmental encrypted flash drive. The final data set will be anonymised, and will subsequently be stored for 5 years following completion of data collection. Data will be held in secured NHS computers. London North West University Healthcare NHS trust will act as the main sponsor for the project. The proposed studies may be subject to inspection and audit by the R&D department at London North West University Healthcare NHS Trust under their remit as sponsor to ensure adherence to GCP and the NHS Research Governance Framework for Health and Social Care. The final data set will be stored securely on NHS computers for 5 years. The storage of anonymised data will comply with the data security standards set by St Marks Hospital, North West London University Healthcare NHS Trust.

Study setting

This single centre study will take place at St Mark's Hospital & Academic Institute, Northwick Park, Harrow, UK. St Mark's Hospital is part of London North West University Healthcare NHS Trust. St Mark's is a specialist colorectal hospital and a tertiary centre for complex colorectal disease. Complex cancer patients are referred from other hospital trusts to St Mark's for expert opinion and subsequent exenteration surgery if it is deemed appropriate

following MDT discussion. Two consultant surgeons undertake exenterative surgery in this unit, surgery is supervised by either one of these experienced consultants although part of the surgical procedure may be undertaken by a post CCT fellow or a senior trainee.
60 patients underwent exenteration surgery at St Mark's over the last year and we are finding our number are increasing year on year therefore completion of recruitment over the two year period would be feasible. The aim would be to commence recruitment in October 2018 and complete recruitment by October 2020. Funding for equipment and resources is possible due to funds held within the BiCyCLE research account. As an academic unit St Mark's Hospital and LNWUH NHS Trust (the Sponsor) has a vast experience of running RCTs and the local R&D at LNWUH NHS will support us in the trial set up and recruitment with access to further expertise from Imperial College London.
Patients will be identified in the outpatient clinic, consented and trained in the preoperative clinic. They will undergo their surgery in the theatre complex shared between St Mark's and Northwick Park Hospital. They will use the NMES device at home following training and then postoperatively on the ITU/HDU and colorectal surgery ward (Frederick Salmon Ward) of St Mark's Hospital. Follow up will take place in the outpatient clinic of St Mark's Hospital.

Sample size and recruitment

Eligibility Criteria

Patients will be identified as suitable by the surgical team in the outpatient clinic of St Mark's Hospital. Eligibility will be based on the inclusion and exclusion criteria described below. Initial contact to take part in the study will be made by the research team. Sample size has been defined by a statistical power calculation performed by a clinical statistician.

Inclusion criteria

- Adults age 18 and above
- Male or female
- Primary or recurrent locally advanced rectal cancer amenable to elective radical exenterative surgery

BiCyCLE NMES – Electric Bike

- ASA grade I-III
- Able and willing to consent
- Participation in other concurrent trials is acceptable – following discussion with trial team of both studies.

Exclusion criteria

- Lack of patient consent
- Wide spread metastases not amenable to curative resection
- Contraindication to NMES (see appendix 6)
- Pre existing neuromuscular degenerative disease
- Participation in other trials where agreement on participation not made in advance by trial teams
- Patients with solitary colon cancer above the level of the peritoneal reflexion which does not require complex pelvic surgery.

SAMPLE SIZE

Sample size has been defined by a statistical power calculation performed by a clinical statistician. Patients meeting the eligibility criteria will be identified by the clinical team and approached, if identified as being a suitable candidate, by the research team. Proposed fields of data collection are listed in Appendix 4.

STATISTICAL ASSESEMENT OF SAMPLE SIZE

The primary objective of the study is to compare standard care and placebo stimulation vs. standard care with active pre-op and postoperative NMES in terms of muscle attenuation (MA) at 3 months post-procedure. Martin and colleagues in their paper suggested a standard deviation of 8.6 HU for males and 10.2 HU for females. Splitting this down the middle we assume an overall SD of 9.4 HU for the group.

Another consideration in the analysis and thus the sample size calculation is that we are planning a measure of MA at baseline. Typically, one would assume some association between baseline value of an outcome, and those at a subsequent time point. i.e. those with higher baseline values are likely to have higher follow-up values. By factoring in the baseline values to the calculation, one can typically get a more precise estimate of the group difference, which in turns results in a lower sample size. The sample size gains depend on how strongly the baseline and follow-up time points are associated. As this is relatively unknown, we have assumed a fairly weak correlation of about 0.3 between the time points.

There are a couple of other statistical quantities to define for the sample size calculation as well, the significance level and power. We have set these to fairly standard values, a 5% significance level and 90% power.

Based on this information, we calculate that to show a difference in MA of 9 units between groups would require a sample size of 21 per group (42 in total).

Given that the possible recruitment targets exceed this we can revise the calculation to detect smaller differences. With 42 in total you might be fairly confident of detecting a difference of 9 HU between groups. However, we might miss out on being able to detect a slightly smaller difference, which might still be of interest. Therefore in order to show a difference in MA of 8 HU we need a sample size of 54 individuals.

To allow for an estimated dropout rate of 5%, 58 patients will be recruited into the study in order for us to power our primary outcome with an equal number of patients in each arm. The dropout rate of 5% is an estimate based on the fact that the treatment period is short and supervised for the most part in hospital. For the primary outcome to be measured we require the pre and post operative CT scans and therefore are not taking into account the potential drop out from the trial out side this time period.

In summary the primary outcome is muscle attenuation at 3 months post-procedure as described above. This will be analysed using analysis of covariance (ANCOVA). The 3 month

value will be considered as the outcome, with the baseline muscle attenuation considered as a covariate.

The sample size is based on a comparison of the primary outcome, muscle attenuation at 3 months post-procedure between the two groups. Previous research (Martin et al) has suggested a standard deviation for this outcome 9.4 HU. A difference in outcome between groups of 8 HU would be regarded as being of clinical importance. An ANCOVA approach to the analysis will be used, with an adjustment for the muscle attenuation values at baseline. A relatively small correlation of 0.3 between the baseline and outcome values is assumed. Factoring in the baseline values, and with a 5% significance level and 90%, it is calculated that 27 patients per group, 54 in total, including the 5% drop out 57 participants are required for the study, we have rounded up to this up to 58 to allow equal numbers in each arm.

Sampling technique

All patients during the two year study period (or until the required number of patients required by the power calculation is met) who are eligible to enter the study will receive an invitation. They will be randomised into either the treatment or control arm as described earlier within the protocol. Eligible patients will be identified by the clinical team and approached by members of the research team.

Recruitment

Patients deemed to meet the eligibility criteria would be identified by the clinical team in the outpatient clinic at St Mark's. The clinical team will alert the research team who in turn will approach the patient. Written information in the form of a participant information sheet (PIS) will be provided at this stage. Patients may wish to decline participation at this stage or discuss further at their forthcoming pre assessment appointment. Consent will take place at the preoperative assessment clinic and will be undertaken by a research team member.

Sample identification

As described above, the clinical team will identify participants to the research team who will in turn approach the patient.

Sample size will be determined with the assistance of a clinical statistician contracted to LNWUH NHS Trust.

Patients will not receive financial remuneration for taking part. No extra hospital visits are required and therefore no travel costs for extra visits will be necessary.

Consent

Informed consent will be obtained prior to the participant undergoing any activities that are specifically for the purposes of this study. Written consent will be taken on the second meeting with the research team member at the patients' second clinic appointment. The consent form will meet the necessary REC, local regulatory requirements and legal requirements.
Patients will receive a REC approved PIS including a sample consent form on their initial visit to the clinic, which will be provided by the research team member. At their next appointment the research coordinator or appointed member of the research team will take written consent. In order to be eligible for the study the participants must have the capacity to consent themselves. Prior to this a full and frank discussion between the potential participant and the research team member, about the nature and objectives of the study and possible risks associated with their participation
The opportunity for potential participants to ask questions will be given at any meeting with the clinical or research team and contact phone numbers and email addresses for the research team will be provided in the PIS.

Withdrawal Criteria

Patients will be withdrawn from the study if they change their mind with regard to consent, or develop symptoms that means they fail to meet the inclusion criteria or suffer unexpected or unforeseen side effects of NMES treatment. Patients are entitled to withdraw from the study at any time without having to give a reason for doing so, and without it affecting their clinical

treatment. This will be made clear to the patients at invitation in the participant information sheet and at the time of consent.

Ethical and regulatory considerations

Assessment and management of risk

All patients taking part in this study will have capacity and would have given informed consent and as such no immediate safeguarding issues are foreseen.

The intervention lasts for a limited period of the study – approximately two weeks preoperatively and four weeks or until patient returns to independent ambulation postoperatively. All other data collected falls in line with routine clinical care. The timeframe which poses the most potential risk or uncertainty will be the early stages of the post operative treatment period, during this time the patient will be supervised and closely monitored in hospital so any potential risks or problems will be identified early and managed appropriately in a safe and timely manner.

Neuro-muscular stimulation will be delivered by a MicroStim Exercise Stimulator MS2v2 (Odstock Medical Limited, Wiltshire, UK) using two self-adhesive electrodes placed on the anterior thigh over the body of the quadriceps muscle and the lower back. This device is CE marked and used regularly in clinical practice in a variety of settings. The device will be used in accordance with the manufacturers instructions. The protocol for usage has been used in numerous previous studies and has been found to be safe (13).

We will not use the device near/on surgical wounds or in areas, which may stimulate the resected tumour, bed as there is a theoretical risk of stimulation of the tumour.

Full training will be given to participants prior to using the device by the members of the research team, (i.e. research fellows, research nurses and research physiotherapists), who in turn have been trained in using the device by the manufacturer or experience team member. Participants may experience a slight reddening of the skin under the electrode pads following use, this is normal and should fade within the hour however if it persists, we advise the participants seek medical advice. The greatest potential risk is pain secondary to muscle contraction, this will be momentary and immediate cessation of device usage should solve this. Patients will increase

the degree of contraction in increments and so it is unlikely the will use an amplitude which would induce significant discomfort. Participants will be advised to always switch the device off prior to removing the pads as there is a theoretical risk of passing a current across the chest if they were to hold an electrode in each hand at the same time with the device switched on. The device should therefore always be switched off prior to connecting or removing the electrodes, this will be made clear to the participants.

If there is any evidence of damage to the device it should not be used, this will be made clear to the patients. The device should not be used near water or surgical diathermy. The device should not be cleaned with spirit cleaning agents except for infection control purposes. Patients must not drive or operate heavy machinery whilst using the stimulator. Patients with contraindications for device use such as those with a pacemaker and those with untreated epilepsy will be excluded from the trial. Should patients have concerns they will have the contact details of the research team.

Research Ethics Committee (REC) and other Regulatory review & report

Regulatory Review & Compliance

The Chief Investigator has obtained approval from the London Queen Square Research Ethics Committee. The Chief Investigator will require a copy of the Trust R&D approval letter before accepting participants into the study. The study will be conducted in accordance with the recommendations for physicians involved in research on human subjects adopted by the 18th World Medical Assembly, Helsinki 1964 and later revisions.

Amendments

Any amendment to the study, the Principle Investigator, in agreement with the sponsor will submit information to the appropriate body in order for them to issue approval for the amendment. For a substantial amendment, the sponsor will submit a valid notice of amendment via IRAS to the REC & HRA for consideration. For a non-substantial amendment, a notification form will be completed and sent directly to the HRA alongside any corresponding documents. The Chief Investigator will

be responsible for the decision to amend the protocol and for deciding whether an amendment is substantial or non-substantial. The Chief Investigator will work with the R&D department at LNWUH NHS trust as well as the R&D department locally so they can put the necessary arrangements in place to implement the amendment to confirm their support for the study as amended. The amendment history will be tracked via Appendix 3 electronically and also a hard copy will be kept in the TMF (Trial Master File) in order to identify the most recent protocol and any other study related documents.

Peer review

Peer review has been sought from experts in colorectal surgery and NMES and appropriate amendments made on their comments and criticisms. Peer reviewers are listed below.

- Dr Tamsyn Street – National Functional Electrical Stimulation Centre, Salisbury NHS Foundation Trust, Salisbury, UK
- Prof Nader Francis – Consultant colorectal surgeon, Yeovil District Hospital NHS Foundation Trust, Yeovil, UK

Patient & Public Involvement

A local patient focus group, has been actively consulted and involved in this study's design, including the development of the satisfaction survey, consent form and participant information leaflet, we also gave the patient group an opportunity to trial the device and feed back to us on its ease of usage. The patient group raised a number of comments and constructive criticisms and we have adjusted the protocol and PIS accordingly. We have included comments raised in appendix 5. There will also be an element of dynamism to the study and should either patients, clinicians or researchers identify design deficiencies then we will adjust the study accordingly, halt recruitment and apply for an amendment if deemed appropriate by the Chief Investigator.

Protocol compliance

Accidental protocol deviations can happen at any time and will be adequately documented on the relevant forms and reported to the Chief Investigator and Sponsor immediately. Deviations from the protocol, which are found to frequently recur, are not acceptable, will require immediate action and could potentially be classified as a serious breach.

Data protection and patient confidentiality

The Chief Investigator will preserve the confidentiality of participants taking part in the study and will work in accordance with the Caldicott Principles, GDPR 2018 and any relevant NHS Trust organisational policies.

The data for analysis in the study will be pseudo-anonymised by the clinical research fellows so that the personal data is not used in the research project. Only the clinical care team will have access to the data during the study. All personal data will be stored in password-protected NHS computers in locked rooms in NHS premises.

The data generated from the study will be reviewed with the clinical team. The data will be stored by LNWH for 15 years following the end of the study.

Indemnity

London North West University Healthcare NHS Trust holds negligent harm and non-negligent harm insurance policies, which apply to this study.

Access to the final study dataset

The anonymity and confidentiality of the information provided for this study will be ensured by de-identification wherein a study ID will be allocated to the patient; only the Chief Investigator and principle investigator will be able to link the study ID to the patient identity and health information, once the data set has been populated the code to the patient ID will be destroyed. This will leave a fully anonymised dataset. Only this dataset will be used in analysis. All of electronic information relating to the study will be stored on a secure password protected database on the Trust NHS computer network and all hardcopy information will be stored under lock and key. Back ups will be performed onto an encrypted flash drive whose specifications meet the standards required by the NHS for confidentiality. This will be stored on the hospital

site under lock and key. These will only be accessible to the primary investigators (i.e.CI and PI). If any information is shared with a third party (e.g., a statistician), no information identifying the patient (e.g., NHS/hospital number, date of birth or name) will be provided to them i.e. fully anonymised.

Study management

The day-to-day management of the study will be co-ordinated through the Principal Investigator Mr Pring and study team including the Principal Investigator & nurse/research coordinator/radiographer/physiotherapist.

Trial Data Monitoring Committee

The Trial Data Monitoring Committee consists of Dr A Stearns (Honorary Senior Clinical lecturer, UEA and Consultant Surgeon Norfolk and Norwich NHS Foundation Trust) and Dr Christos Kontovounisios (Senior Clinical Lecturer and Honorary Consultant Surgeon, Imperial College London). They will provide independent assessment of the safety, scientific validity and integrity of clinical trial as it progresses. They will be bound by terms of reference set out by the sponsor.

Dissemination policy

Dissemination policy

Results will be submitted to a peer reviewed journal for publication and presented at conferences. Work from this study will also form part of a Doctoral Thesis, which will be retained by Imperial College London. Patients and data will be anonymised.

Authorship eligibility guidelines and any intended use of professional writers

The rights of authorship will be held by Mr Jenkins and Mr Pring, Individuals who make a significant contribution to the study either with data collection or analysis will be recorded as

contributing authors. The intellectual property resulting from the study will belong to Messrs Jenkins, Pring and Malietzis.

The work from this study may form the subject of a Chapter of a Doctoral Thesis by Mr Pring, which will be presented to Imperial College London for defence. The work may also be published in peer reviewed scientific journals and presented at international, national or local scientific conferences and meetings.

Publication Policy

The study findings will be published in peer-reviewed scientific journals, and presented at local, national and international meetings and conferences. Future researchers at St Mark's will be able to access a summary of information through the organisational website of St Mark's Hospital & Academic Institute. Data collected in this research database will not be shared with other individuals or research groups. Any future decision to share such data will require an application to and approval from an NHS Research Ethics Committee as part of a substantial amendment to study protocol.

Participants will be made aware of the publication policy before signing consent, and may request to be notified of publications resulting from the data collected in this study.

REFERENCES

1. Malietzis G, Currie AC, Athanasiou T, Johns N, Anyamene N, Glynne-Jones R, et al. Influence of body composition profile on outcomes following colorectal cancer surgery. Br J Surg. England; 2016 Apr;103(5):572–80.
2. Cespedes Feliciano EM, Kroenke CH, Meyerhardt JA, Prado CM, Bradshaw PT, Kwan ML, et al. Association of Systemic Inflammation and Sarcopenia With Survival in Nonmetastatic Colorectal Cancer. JAMA Oncol [Internet]. 2017;94612:e172319. Available from: http://oncology.jamanetwork.com/article.aspx?doi=10.1001/jamaoncol.2017.2319
3. Keller C, Keller P, Giralt M, Hidalgo J, Pedersen BK. Exercise normalises overexpression of TNF-α in knockout mice. Biochem Biophys Res Commun. 2004;321(1):179–82.
4. Starkie R, Ostrowski SR, Jauffred S, Febbraio M, Pedersen BK. Exercise and IL-6 infusion inhibit endotoxin-induced TNF-alpha productions in humans. FASEB J. 2003;17(8):884–6.
5. Jones S, Man W, Gao W, Higginson I, Wilcock A, Maddocks M. Neuromuscular electrical stimulation for muscle weakness in adults with advanced disease (Review). Cochrane Database Syst Rev. 2016;(10).
6. Maffiuletti NA. Physiological and methodological considerations for the use of neuromuscular electrical stimulation. Eur J Appl Physiol. 2010;110(2):223–34.
7. Thompson W, Gordon N, Pescatello L. ACSM's guidelines for exercise testing and prescription. 8th Ed London Lippincott Williams Wilkins. 2010;
8. Binder-Macleod SA, Snyder-Mackler L. Muscle fatigue: clinical implications for fatigue

assessment and neuromuscular electrical stimulation. Phys Ther. United States; 1993 Dec;73(12):902–10.

9. National Clinical FES Centre - Salisbury NHS Foundation Trust [Internet]. [cited 2018 Jan 9]. Available from: http://www.salisbury.nhs.uk/INFORMATIONFORPATIENTS/DEPARTMENTS/CLINICALSCIENCEANDENGINEERING/Pages/NationalClinicalFESCentre.aspx
10. Hasegawa S, Kobayashi M, Arai R, Tamaki A, Nakamura T, Moritani T. Effect of early implementation of electrical muscle stimulation to prevent muscle atrophy and weakness in patients after anterior cruciate ligament reconstruction. J Electromyogr Kinesiol [Internet]. Elsevier Ltd; 2011;21(4):622–30. Available from: http://dx.doi.org/10.1016/j.jelekin.2011.01.005
11. Burke D, Gorman E, Stokes D, Lennon O. An evaluation of neuromuscular electrical stimulation in. Clin Respir J. 2014;407–20.
12. Maddocks M, Lewis M, Chauhan A, Manderson C, Hocknell J. Randomized Controlled Pilot Study of Neuromuscular Electrical Stimulation of the Quadriceps in Patients with Non-Small Cell Lung Cancer. J Pain Symptom Manage [Internet]. Elsevier Inc; 2009;38(6):950–6. Available from: http://dx.doi.org/10.1016/j.jpainsymman.2009.05.011
13. Maddocks M, Halliday V, Chauhan A, Taylor V, Nelson A, Byrne A, et al. Neuromuscular Electrical Stimulation of the Quadriceps in Patients with Non-Small Cell Lung Cancer Receiving Palliative Chemotherapy : A Randomized Phase II Study. PLoS One. 2013;8(12):1–8.
14. Chen BB, Shih TTF, Hsu CY, Yu CW, Wei SY, Chen CY, et al. Thigh muscle volume predicted by anthropometric measurements and correlated with physical function in the older adults. J Nutr Health Aging. 2011;15(6):433–8.

Appendices

Appendix 1- a summative list of submitted documentation

- **Chief Investigator summary CV**
- **Clinical Research Fellow (CRF)/Research Coordinator summary CV**
- **ED-5Q-5L and EORTC QLQ – CR29 Quality of Life Questionnaires**
- **Patient NMES device satisfaction questionnaire**
- **Peer reviewer assessment forms(2 for each reviewer)**
- **Participant information sheet**
- **Consent form**
- **Manufacturers Instruction Sheet MicroStim 2v2**
- **MicroStim CE declaration**
- **Schedule of Events**
- **Statement of Activities**
- **GP Information letter**
- **Placebo arm instruction sheet**
- **Treatment arm instruction sheet**

Appendix 2 – Study flowchart with typical patient time points

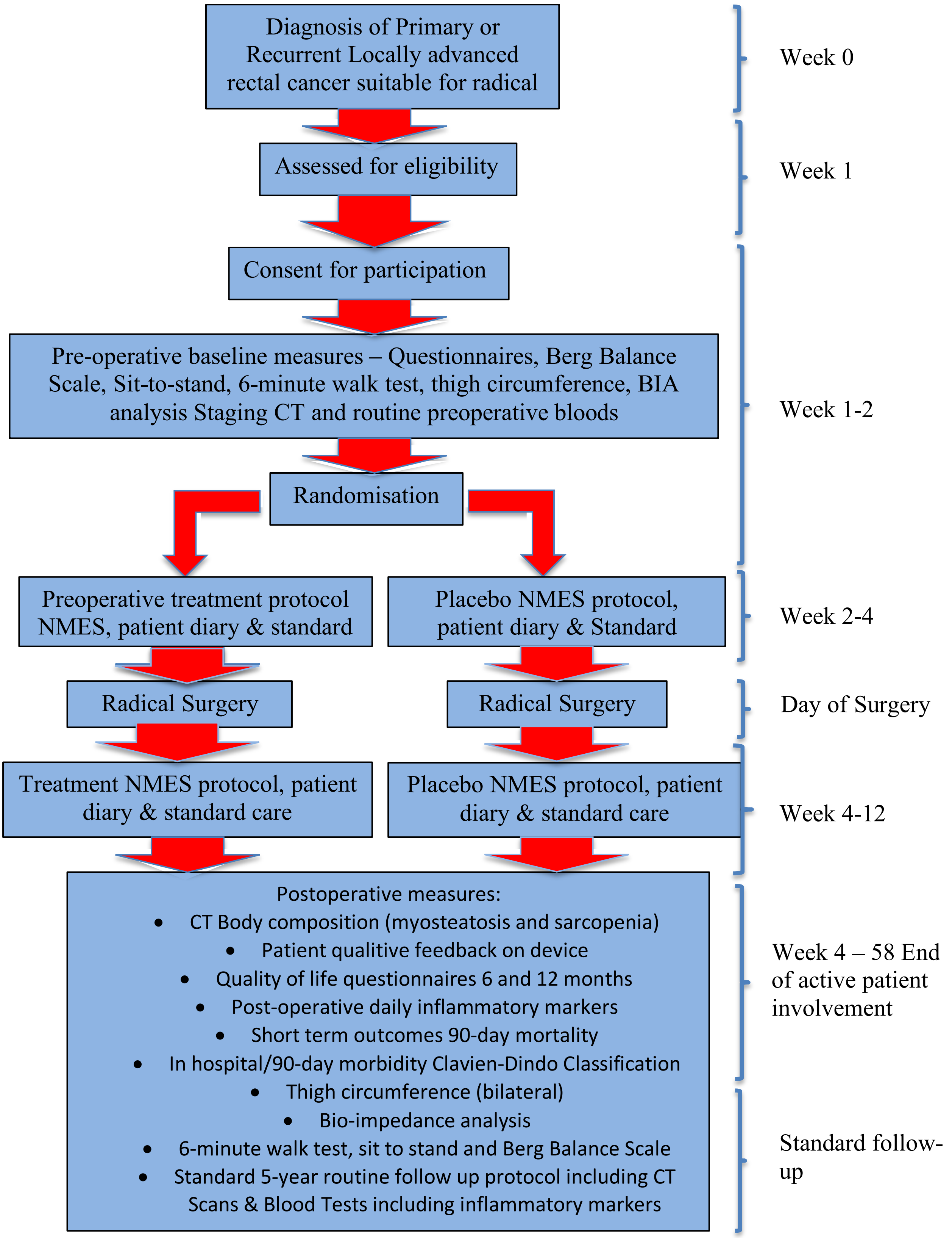

Amendment No.	Protocol version no.	Date issued	Author(s) of changes	Details of changes made
SA1019	5.0	05/04/19	E T Pring	Microstim activity reader data added to secondary outcomes in protocol and information pertaining to this added to the PIS
SA2020	6.0	05/06/20	E T Pring	• Increased recruitment from 57 to 58 patients (to provide equal numbers in each trial arm)<br>• Change of Sponsor representative (due to retirement) Dr A Warnes replaced by Mr Simon Lewis LNWUH Research Governance Manager<br>• Addition of Trial Data Monitoring Committee – Dr A Stearns (Consultant Surgeon Norfolk and Norwich NHS Foundation Trust) and Dr Christos Kontovounisios (Senior Clinical Lecturer and Honorary Consultant Surgeon Imperial College London)

Appendix 4 – Data capture fields

Demographics

- Patient study code
- Patient age
- Patent birth year
- Patient gender
- Patient ethnicity coding
- Weight
- Height
- BMI (Body Mass Index – the body mass divided by the square of the body height)
- Smoking status
- Disease

BiCyCLE NMES – Electric Bike

- ASA grade (American College of Anaesthesiology physical status classification: Grade 1 to 4: 1 No systemic disease, 2 mild systemic disease, 3 severe systemic disease, 4 severe systemic disease which is a constant threat to life)
- Drug history (current medications the patient is taking)
- Past medical history (Other or previous illnesses and surgery)
- Type of cancer (primary/recurrent cancer, location and subtype on histology)
- Grade of Cancer (microscopic level of cellular change)
- Stage of Cancer (CT/MRI/Histology - local level, lymph node gland spread, distant spread)
- Metastases and location (Distant spread – secondary's)
- Number of Lymph nodes (amount of spread to lymph node glands)
- Sentinel node involved (cancer within the nearest node to the main cancer)
- Details of Surgery
- Time to passage of flatus/faeces into the stoma bag following surgery
- Chemotherapy
- Radiotherapy
- CT scan analytical data of Body Composition at the level of the L3 vertebra (specific level of spine in the lower back – chosen to ensure standard measurements across the group)
- Disease Outcomes including 30 day survive
- Blood result markers of Systemic inflammation and haematological/biochemical markers – C-reactive protein (a protein molecule which rises in inflammation), white cell count (blood cells which increase in inflammation and infection), serum albumin (a protein in blood)
- Outcome death / recurrence of cancer / disease free survival
- Quality of Life as per validated questionnaire ED-5Q-5L & EORTC QLQ – CR29
- Qualitative data on the tolerability and participant experience of the device
- CPET
- Patient compliance diary data
- Bio-impedance analysis and anthropometric measurements

Appendix 5 – Patient group comments and criticisms (PPI representatives BG, LG, LJ, TW & RM)

The project was generally well received by the patient group, they thought it sounded interesting and agreed that if there are ways in which patients can promote ownership and autonomy – "giving them some control" over their condition in hospital then this was generally a good thing. There were a number of specific comments, which we have addressed below.

- Relating to study aims we stated "evidence suggests that patients with better muscle mass and quality do better after surgery" one of our patient representatives commented "Is this a fact?". We have therefore rephrased this as "there is growing evidence from a number of published studies that patients with better muscle mass and quality do better after surgery."

BiCyCLE NMES – Electric Bike

- Study aims: we stated, "[the NMES] can deliver impulses which bring about repetitive muscle contractions mimicking physical exercise" One of our patients commented "e.g. walking etc." We have therefore added examples of the type of exercise mimicked here "e.g. resistance exercise such as lifting a weight or load"

- Relating to the PIS section 2, which reads. "Allocation will be on a random basis but you will be told which group you have been allocated to". Patient representative underlined as shown with comment: "What difference – like for like in each group (measurements before and after)?" We have therefore clarified that only the intervention is different, that each patient in each group will be randomly assigned – assignment will not be based on individual characteristics. The measurements taken in each group will be the same and the follow up process will be identical in each group.

- Relating to PIS section 2 standard group section: "you will receive the standard care any patient receives following your type of surgery i.e. the same care a patient would receive if not in the trial." Patient rep comment: "Physio with trained staff". We have therefore made it clear in the PIS that both those in the standard care and treatment group will both receive physiotherapy performed by trained staff and that this will not differ between groups nor will it differ for those patients who decide not to be in the trial.

- Relating to PIS section 2 "both groups" section: "we may analyse your blood looking at extra commonly tested parameters". Patient comment :"such as…". We have therefore clarified this by adding "such as inflammatory proteins e.g. C-reactive protein and nutritional markers e.g. vitamin levels, albumin and salts/electrolytes".

- We discussed the quality of life questionnaires in section 2 of the PIS this is reiterated in section 3 on risks and disadvantages where we state "there is a small chance answering questions will cause psychological distress" One patient reviewer commented "is this a disadvantage or a real risk"? We have therefore rephrased this saying "some of the questions within the quality of life surveys are of a personal or sensitive nature if these questions cause you any distress or concern you are under no obligation to answer them."

- There were several comments relating to repetition of information e.g. section 4 Comment "repeat from para 1"; "you will be asked to repeat 3 questionnaires over a year" section 2 and You will be asked to answer questions about you quality of life at three time points" section 3. We however do feel this reiteration is important, does not cause confusion but confirmation and there is no real disadvantage to its inclusion we therefore it was agreed with the PPI group to keep these in the PIS.

BiCyCLE NMES – Electric Bike

- Section 10 "BiCyCLE… trial group at St Mark's Hospital and Imperial College London headed by Mr Ian Jenkins" Comment: "is this Mr John Jenkins"? We have therefore clarified this by writing "…Mr Jenkins (Consultant Surgeon and lead for complex rectal cancer)."

- Section 12 PIS "always remember to turn off the stimulator before you remove the electrodes" Patient comment "this should be in the do's and don't section."

- Patients were happy with the consent form. They liked the layout of the PIS, they liked the consent form attached to and continuous with the PIS.

- The trial specific instruction flyer was well received one suggestion was that at point 3 on the flyer starting "unpeel the electrodes" it was suggested we simplified this by saying "do not pull the pads off by their wires". We have added this to the instruction leaflet.

- They liked the instruction leaflet designed specifically for the study, they liked that it was in colour for ease of reading, one patient advisor did ask that we "include the diagram from the instruction manual on line as this is very clear and really useful" We have therefore included this diagram and sought permission from Dr Steve Crook at OML to use it in the flyer. Patients will all also receive a copy of the MicroStim 2v2 instruction manual if they are in the intervention group.

Appendix 6 – Contraindications and cautions of NMES

The MS2v2 is **not** to be used by people who have implanted electronic devices (pacemakers etc.) unless under specialised medical supervision.

Persons with uncontrolled epilepsy.

The safety of electrical stimulation in pregnancy has not been determined and therefore contraindicated in this study.

Some high level spinal cord lesion patients may suffer autonomic effects after or during electrical stimulation.

BiCyCLE NMES – Electric Bike
