# Supplementary material for: BiCyCLE NMES—neuromuscular electrical stimulation in the perioperative treatment of sarcopenia and myosteatosis in advanced rectal cancer patients: design and methodology of a phase II randomised controlled trial
Source: Trials. 2021 Sep 15;22:621. doi: 10.1186/s13063-021-05573-2 (PMC8442432; doi:10.1186/s13063-021-05573-2)
Supplement: Supplementary file 2 — Additional file 2. [file 13063_2021_5573_MOESM2_ESM.pdf]

Trial Title: Body Composition Manipulation in Colorectal Cancer (BiCyCLE): Neuro-Muscular Electrical Stimulation (NMES) and its effect on the systemic inflammatory response and changes in body composition following radical surgery for locally advanced rectal cancer **[BiCyCLE NMES]**

ClinicalTrials.gov Identifier: NCT04065984

Trial Sponsor: London North West University Healthcare NHS Trust

Trial Specific Independent Data Monitoring Committee (IDMC) Charter

Version 1.0 Date 05/07/20

IRAS Number: 242002

SPONSORS Number:
RD18/115

FUNDERS Number: N/A
REC Reference:
19/LO/0259

Authorised by:

Name: Mr J T Jenkins Role: Chief Investigator
Signature: Date: 05/07/20

Prepared by:

Name: Mr E T Pring Role: Principle Investigator and Trial Coordinator
Signature: Date: 05/07/20

CONTENT	CHARTER DETAILS
**1. INTRODUCTION**	
Name of trial, ISRCTN and/or EUDRACT number and Sponsor name	Insert Body Composition Manipulation in Colorectal Cancer (BiCyCLE): Neuro-Muscular Electrical Stimulation (NMES) and its effect on the systemic inflammatory response and changes in body composition following radical surgery for locally advanced rectal cancer<br>ClinicalTrials.gov Identifier: NCT04065984
Objectives of trial, including interventions being investigated	Does pre and post-operative neuro muscular electrical stimulation decrease myosteatosis, increase muscle mass, decrease the systemic inflammatory response and improve short and long term outcomes in patients undergoing radical surgery for primary or recurrent locally advanced rectal cancer?
Outline of scope of charter	The purpose of this document is to describe the membership, terms of reference, roles, responsibilities, authority, decision-making of the IDMC for the above trial, including the scope of meetings, methods of providing information to and from the IDMC, frequency and format of meetings, statistical issues and relationships with other committees.
**2. ROLES AND RESPONSIBILITIES**	
A broad statement of the aims of the committee	To protect and serve patients of the BiCyCLE NMES Trial (especially re: safety) and to assist and advise Chief Investigator(s)(CIs) and Trial Management Group (TMG) to protect the validity and credibility of the BiCyCLE NMES Trial<br>To safeguard the interests of the BiCyCLE NMES Trial participants, assess the safety and efficacy of the interventions during the trial, and monitor the overall conduct of the BiCyCLE NMES Trial.
Terms of reference	The IDMC should receive and review the progress and accruing data of the BiCyCLE NMES Trial and provide advice on the conduct of the trial to the Trial Team<br>The IDMC should inform the Chair of the IDMC if, in their view:<br>(i) the results are likely to convince a broad range of clinicians, including those supporting the trial and the general clinical community, that the trial arm, or the participants/patients, is clearly indicated or contraindicated, and there is a reasonable expectation that this new evidence would materially influence patient management; **or**<br>(ii) it becomes evident that no clear outcome would be obtained.
Specific roles of IDMC	Interim review of the trial's progress including updated figures on recruitment, data quality, and main endpoints

CONTENT	CHARTER DETAILS
	including safety data.<br>A selection of specific aspects could be compiled from the following list:<br>• assess data quality, including completeness to encourage collection of high quality data<br>• monitor recruitment figures and drop-outs to follow-up<br>• monitor compliance with the protocol by participants and investigators<br>• monitor trial/project conduct – organisation and implementation of trial protocol (the IDMC should only perform this role in the absence of other trial oversight committees)<br>• monitor evidence for treatment differences in the main efficacy endpoints<br>• monitor evidence for treatment harm (eg toxicity data, SAEs, deaths)<br>• review the report of suspected unexpected serious adverse reaction (SUSAR) provided by the trial team whenever occurs<br>• decide whether to recommend that the trial continues to recruit participants or whether recruitment should be terminated either for everyone or for some treatment groups and/or some participant subgroups<br>• suggest additional data analyses<br>• advise on protocol modifications (e.g. inclusion criteria, trial endpoints, or sample size)<br>• monitor planned sample size assumptions, preferably with regards to assumptions about the control arm outcome<br>• monitor continuing appropriateness of patient information<br>• monitor compliance with previous IDMC recommendations<br>• consider the ethical implications of any recommendations made by the IDMC<br>• assess the impact and relevance of external evidence<br>• maintain confidentiality of all trial information that is not in the public domain<br>• protect validity and scientific credibility of the trial
**3. Before or early in the trial**	
Whether the IDMC will have input into the protocol	All potential IDMC members should have sight of the protocol/outline (current versions & superseded versions if requested) before agreeing to join the committee. The IDMC is being proposed after the trial has already begun and scrutiny by a research ethics committee, Health Research Authority and/or Medicines and Healthcare products Regulatory Agency (MHRA) has already taken place. IDMC members should be independent and

CONTENT	CHARTER DETAILS
	constructively critical of the ongoing trial, but also supportive of aims and methods of the trial.
Any specific regulatory issues	The *BiCyCLE NMES Trial has been approved by the HRA, Queens Square REC* and local R&D (Reference numbers above). There are no other specific regulatory issues.
Any other issues specific to the treatment under study	NMES and the microstim 2v2 device are currently approved for clinical use in the UK.
Whether members of the IDMC will have a contract	IDMC members do not formally sign a contract but formally register their assent to join the group by confirming (1) that they agree to be on the IDMC and (2) that they agree with the contents of this Charter. Any competing interests should be declared at the same time. Members should complete and return the form Annexe 1. Observers attending any part of the meeting should sign a confidentiality agreement on the first occasion they attend all or part of a meeting (Annexe 2).
**4. Composition**	
Membership and size of the IDMC	The members are not be involved with the trial in any other way nor have competing interests that could impact on the trial. All declaration of interests or complete disclosure should be declared by members of the committee. A short competing interest form should be completed and returned by the IDMC members to the trial coordinating team (Annexe 1).<br>The members of the IDMC for this trial are:<br>(1) *Mr Adam Stearns*<br>(2) *Mr Christos Kontovounisios*
The responsibilities of the trial coordinating team	The trial coordinator/or project manager of trial will assist the trial to produce the non-confidential sections of the IDMC report. The trial coordinator/or project manager may attend open sessions of the meeting.
The responsibilities of the CI and other members of the TMG	The CI, may be asked, and should be available, to attend open sessions of the IDMC meeting.
**5. Relationships**	
Clarification of whether the IDMC are advisory (make recommendations) or executive (make decisions)	The IDMC does not make decisions about the trial, but rather makes recommendations to the trial team/committee.
**6. Organisation of IDMC meetings**	
Expected frequency of IDMC meetings	The exact frequency of meetings will depend upon any statistical plans specified and on trial events. It is

**CONTENT**	**CHARTER DETAILS**
	recommended that the IDMC meet at least yearly.<br>An unplanned IDMC meeting may be called by the Chair if there is an emergency concern regarding the safety of participants.
Whether meetings will be face-to-face or by teleconference	It is recommended that all meetings should be virtual/face-to-face if possible.
**7. TRIAL DOCUMENTATION AND PROCEDURES TO ENSURE CONFIDENTIALITY AND PROPER COMMUNICATION**	
Intended content of material to be available in open sessions	<u>Open sessions</u>: Accumulating information relating to recruitment and data quality (eg data return rates, sample collection, treatment compliance) will be presented. and Total numbers of events for the primary outcome measure and other outcome measures may be presented, at the discretion of the IDMC.
Intended content of material to be available in closed sessions	<u>Closed sessions</u>: In addition to all the material available in the open session, the closed session material will include efficacy and safety data by treatment and placebo group.
The people who will see the accumulating data and interim analysis	DMC members do **not** have the right to share confidential information with anyone outside the IDMC, including the CI.
Responsibility for identifying and circulating external evidence (eg from other trials/ systematic reviews)	The CI, the trial coordinating team will collate any external evidence and information for the presentation in an open session as asked by IDMC.
To whom the IDMC will communicate the decisions/ recommendations that are reached	The IDMC will report its recommendations in writing to the to the trial team or sponsor's representative. This should be copied to the trial statistician (or trial coordinator).<br>In its communications, the IDMC should be careful not to relay any unnecessary information to the Trial Team.
What will happen to the confidential papers after the meeting	The IDMC members should store the papers safely after each meeting so they may check the next report against them. After the trial is reported, the IDMC members should destroy all interim reports.
**8. DECISION MAKING**	
What decisions/recommendations will be open to the IDMC	Possible recommendations could include:<br>• No action needed, trial continues as planned<br>• Early stopping due, for example, to clear benefit or harm of a treatment, futility, or external evidence

CONTENT	CHARTER DETAILS
	• Stopping recruitment within a subgroup<br>• Extending recruitment (based on actual control arm response rates being different to predicted rather than on emerging differences) or extending follow-up<br>• Stopping a single arm of a multi-arm trial<br>• Sanctioning and/or proposing protocol changes
The role of formal statistical methods, specifically which methods will be used and whether they will be used as guidelines or rules	This Charter should include or provide reference to the planned interim analyses and statistical guidelines, ie the IDMC should review and agree any interim analysis plan.
How decisions or recommendations will be reached within the IDMC	Issues to be specified can include:<br>• The decision making methods and criteria that will be adopted for guiding deliberations<br>• The process of decision making, including whether there will be voting or other formal methods of achieving consensus. The method of deliberation should not be revealed to the overseeing committee as this may reveal information about the status of the trial's data<br>• The role of the Chair - to summarise discussions and encourage consensus; it may be best for the Chair to give their own opinion last.
Can IDMC members who cannot attend the meeting input	If the report is circulated before the meeting, IDMC members who will not be able to attend the meeting may pass comments to the IDMC Chair for consideration during the discussions.
**9. REPORTING**	
To whom will the IDMC report their recommendations/decisions, and in what form	Usually, this will be a letter to the Trial Team or Sponsor's representative. A timescale should be specified eg usually within 3 weeks. A copy of the IDMC recommendation will be stored in the trial master file.
Whether minutes of the meeting be made and, if so, by whom and where they will be kept	These details should be specified (separate records may be required for open and closed sessions). The IDMC members should sign off any minutes or notes.
What will be done if there is disagreement between the IDMC and the body to which it reports	Specify which committee has primacy or how disagreement will be resolved, eg a further committee may be convened to adjudicate.
**10. AFTER THE TRIAL**	
Publication of results	At the end of the trial there may be a meeting to allow the IDMC to discuss the final data with the key members of

**CONTENT**	**CHARTER DETAILS**
	Trial Team and give advice about data interpretation.<br>The IDMC may wish to see a statement that the trial results will be published in a correct and timely manner.
The information about the IDMC that will be included in published trial reports	IDMC members should be named and their affiliations listed in the main report, unless they explicitly request otherwise. A brief summary of the timings and conclusions of IDMC meetings should be included in the body of this paper.
Whether the IDMC will have the opportunity to approve publications, especially with respect to reporting of any IDMC recommendation regarding termination of a trial	The IDMC will be given the opportunity to read and comment on publications before submission.

Annexe 1: Agreement and potential competing interests form

BiCyCLE NMES

Please complete the following document and return to the BiCyCLE NMES Trial Co-ordinator.

(Check box to agree)

☐ I have read and understood the IDMC Charter version 1.0, dated 05/07/20

☐ I agree to join the IDMC for this trial

☐ I agree to treat all sensitive trial data and discussions confidentially

The avoidance of any perception that members of an IDMC may be biased in some fashion is important for the credibility of the decisions made by the IDMC and for the integrity of the trial.

Possible competing interest should be disclosed. In many cases simple disclosure up front should be sufficient. **Table 1** lists potential competing interests.

☐ **No,** I have no competing interests to declare
☐ **Yes,** I have competing interests to declare (please detail below)

Please provide details of any competing interests:

____

____

____

Name: ____________________________

Signed: ___________________________ Date: ______________

Annexe 2: Agreement and confidentiality agreement for observers

Insert trial name

Please complete the following document and return to the BiCyCLE NMES Trial Co-ordinator.

(please check box to agree)

☐	I have received a copy of the IDMC Charter version 1.0, dated 05/07/20
☐	I agree to attend the IDMC meetings either face to face or teleconference
☐	I agree to treat as confidential any sensitive trial information gained during this meeting unless explicitly permitted

Name: ___________________________

Signed: __________________________ Date: ______________

Annexe 3: BiCyCLe NMES Trial Contacts

Contacts

Chief Investigator

Mr J T Jenkins
St Marks Hospital, Northwick Park, Watford Road, Harrow, HA1 3UJ
i.jenkins@nhs.net

Principle investigator and Trial Coordinator

Mr E T Pring
St Marks Hospital, Northwick Park, Watford Road, Harrow, HA1 3UJ
edward.pring@nhs.net

Trial Statistician

Mr Paul Bassett

paul@statsconsultancy.co.uk
