# Supplementary material for: BiCyCLE NMES—neuromuscular electrical stimulation in the perioperative treatment of sarcopenia and myosteatosis in advanced rectal cancer patients: design and methodology of a phase II randomised controlled trial
Source: Trials. 2021 Sep 15;22:621. doi: 10.1186/s13063-021-05573-2 (PMC8442432; doi:10.1186/s13063-021-05573-2)
Supplement: Supplementary file 3 — Additional file 3. [file 13063_2021_5573_MOESM3_ESM.pdf]

BiCyCLE NMES Participant Information Sheet

**Study title:**	**BiCyCLE NMES: Electric Bike Trial**: A study to examine the effects of neuro-muscular electrical stimulation on body composition following major surgery for locally advanced rectal cancer – a single centre double blind randomised control trial	
**Study Sponsor**	London North West University Healthcare NHS Trust	
**Chief investigator**	Mr John T Jenkins<br>Consultant Surgeon and Complex Cancer Lead,<br>Department of Surgery,<br>St Marks Hospital, London North West University Healthcare NHS Trust, Harrow, HA1 3UJ	Telephone:<br>020 8869 4177<br>i.jenkins@nhs.net
**Principle Investigator & Trial Coordinator**	Mr Edward T Pring,<br>George Davies Surgical Research Fellow,<br>Imperial College London and St Mark's Hospital, London North West University Healthcare NHS Trust, Harrow, HA1 3UJ	Telephone:<br>020 8869 4177<br>e.pring17@imperial.ac.uk

Introduction

We would like to invite you to take part in our research study. Before you decide whether or not you would like to take part, we would like you to understand why the research is being done and what it would involve for you. We will go through this information sheet with you and answer any questions you may have. We suggest this should take about 30 minutes. Please read this information sheet carefully and if you wish to talk with relatives or friends, before deciding whether or not to participate.

If you decide to participate we thank you. Please be advised if you decide not to take part there will be no disadvantage to you or your clinical care.

What is the aim of this study?

When you are unwell, especially with an illness such as bowel cancer you lose muscle as part of the disease process. After an operation, because you are not as mobile as you would be at home you also suffer muscle wasting becoming less strong. There is growing evidence from a number of published studies that patients with better muscle mass and quality do better after surgery. Mr Jenkins and his team are therefore looking at ways we can try and prevent or reduce this muscle loss and therefore improve patient outcomes.

A neuro-muscular electrical stimulator (NMES) is a small portable battery powered device, which can deliver impulses, which bring about repetitive muscle contractions mimicking physical exercise such as walking up a hill, cycling or lifting a moderate weight or load. The aim of this project is to see whether using a NMES portable electrical stimulator before and after surgery helps maintain your muscles and stops them wasting away and in doing so improves your outcome.

1. **Why have I been chosen?**

This study is recruiting those aged 18 and over with resectable (i.e. operable) locally advanced rectal cancer who will receive major surgery to the pelvis, known as an exenteration, to remove the tumour. We are not recruiting people with inoperable tumours or people who have secondary deposits (metastases), which cannot be removed by surgery. If you choose not to be recruited, your data will not be analysed for this research.

2. What will happen to me if I take part?

If you choose to take part in this research, we will allocate you to either the electrical stimulator “NMES treatment group” or the “NMES Placebo group”. Allocation will be on a random basis performed by specialist computer randomisation software and you will not be told which group you have been allocated to. Once allocated you will not be able to change to the other group within the trial. It is important to note only the intervention is different, assignment to a specific group will be entirely random and not be based on individual characteristics. The measurements taken in each group will be the same and the follow up process will be identical in each group.

When using the NMES device some people have a noticeable contraction while others do not. Some people are aware of a sensation whilst others are not. People who experience such a sensation quickly become use to it.

<u>Treatment and Placebo NMES</u>

If you are allocated to the treatment or placebo NMES group you will be trained to use the NMES device during your clinic appointment, you will be asked to use the device daily as prescribed from the preoperative assessment appointment to the day of your operation. After surgery you will be asked to use the NMES device daily as prescribed for four weeks after your surgery. At this time we will ask you to complete a short questionnaire where you can tell us about your experience of using the device, how convenient it was for you and whether we can improve things in the future for other patients. You will also receive the standard care any patient receives following your type of surgery including physiotherapy by trained staff i.e. this is the same care that a patient would receive if not in the trial.

The rest of your treatment will be the same as the standard treatment for any patient, we may analyse your blood looking at extra commonly tested parameters (e.g. blood counts, inflammatory protein markers, vitamin levels, salts and electrolytes) but this will not affect your care or standard of treatment, we will not require extra blood samples for these tests. The investigators will access your disease related health information such as your blood tests and CT Scan results for analysis.

You will be asked to complete 3 questionnaires over the next year to assess your quality of life before and after surgery. You will be asked about your quality of life at baseline before surgery; you will then be asked about your quality of life at 6 months and 12 months following your operation.

If you choose not to take part in the study, the care provided to you will not be changed or prejudiced due to this decision; this is a purely voluntary study. There is no reimbursement

for time or expenses. All information and data will be anonymised by the research team to ensure your confidentiality is maintained. You may withdraw from the study at any point; there is no need for you to justify your decision.

3. **What happens after the treatment period of ten weeks?**

Following completion of the ten-week trial period no further neuromuscular electrical stimulation therapy will take place, we will ask you to return the device to the research team and complete the device satisfaction questionnaire. Unfortunately, we are unable to offer continued treatment after the trial.

4. **Are there any other disadvantages or risks from taking part in this study?**

Using the NMES may feel unusual to start with as it causes your muscles to contract but this should not be painful. If you do suffer significant discomfort please stop using the device and contact the research team. There have been reports of a slight reddening of the skin or occaisionally minor skin irritation from the conductive pads applied to the skin. This is temporary and usually resolves within an hour once you stop using the device. Please contact the research team if the irritation persists.
You will be asked to answer questions about your quality of life at three time points after your diagnosis of rectal cancer; some of the questions within the quality of life surveys are of a personal or sensitive nature if these questions cause you any distress or concern you are under no obligation to answer them. We will be on hand to advise if you have any queries questions or worries about your care in relation to the trial. All other investigations and treatment will be part of the standard care any patient would receive following this type of surgery. If any other risks or adverse effects become apparent during the course of the study, you will be duly informed.

5. **What should I do if I feel upset or distressed during the study or following either my diagnosis or treatment?**

The Macmillan colorectal cancer nursing team at St Mark's is here to support you through your entire cancer journey. Our experienced team led by Claire Taylor are contactable during working hours and can answer any questions, fears or concerns you may have. You would have been given their contact details following your diagnosis but as a reminder the contact details can be found at the bottom of this information sheet. If you feel you are getting upset

whilst completing the questionnaires you must not feel compelled to complete the questionnaire further. If you have a problem out of hours please contact either your GP or in an emergency your surgical team via the main hospital phone number.

6. What are the alternatives for treatment?

If you choose not to take part in the study there will be no impact on your clinical care, you will receive the standard care, occasionally we use NMES as part of standard care at a treatment level and you may still be offered this treatment if your clinical team feel it may benefit you.

7. What specimens, data or information will be collected, and how will they be used?

The results from biopsies and surgical specimens that would normally be collected as part of your usual care will be collected, but no biopsies or surgical specimens will be collected as part of the study in itself.
It is possible that data generated in this study, but not reported, will be made available for use in future research (e.g. for inclusion in an individual data meta-analysis). If this happens, it will be ensured all data will be provided in a purely de-identified manner.

8. What is the purpose of the diary and how should I complete it?

As part of the study we hope to get an impression of how acceptable and usable the device is for you and how often you and other patients are using it. We can also work out what is the best regime of device usage based on how often patients use it and how their CT scans look after surgery. If you are not using it frequently it is good for us to know as it may help us explain unusual looking results when we analyse the study findings. We would be grateful if you could complete the diary each day even if you don’t use the device. If you miss a day do not worry you can either complete it from what you remember doing or leave it blank. The diary is quick and easy to complete and we will go through how to complete it in detail when we go through you initial training session with the device.

9. Will my taking part in this study be kept confidential?

The anonymity and confidentiality of the information you provide for this study will be ensured by a process called de-identification wherein a study ID is allocated to you; only the primary investigators will be able to link your study ID to your identity and health information.

All of your electronic information relating to the study will be stored on a secure password protected database and all hardcopy information will be stored under lock and key. These will only be accessible to the primary investigators. If any information is shared with a third party (e.g., a statistician), no information identifying you (e.g., NHS/hospital number, date of birth or full name) will be provided to them.

10. Your personal data and General Data Protection Regulation

London North West Healthcare University NHS Trust is the sponsor for this study in the United Kingdom. We will be using information from you and your medical records in order to undertake this study and will act as the data controller for this study. This means that we are responsible for looking after your information and using it properly. London North West Healthcare University NHS Trust will keep identifiable information about you for 5 years after the study has finished.

Your rights to access, change or move your information are limited, as we need to manage your information in specific ways in order for the research to be reliable and accurate. If you withdraw from the study, we will keep the information about you that we have already obtained. To safeguard your rights, we will use the minimum personally-identifiable information possible.
You can find out more about how we use your information by following this link: https://www.lnwh.nhs.uk/hidden/privacy-policy/

London North West Healthcare University NHS Trust will use your name, NHS number and contact details to contact you about the research study, and make sure that relevant information about the study is recorded for your care, and to oversee the quality of the study. Individuals from London North West Healthcare University NHS Trust and regulatory organisations may look at your medical and research records to check the accuracy of the research study. The only people in London North West Healthcare University NHS Trust who will have access to information that identifies you will be people who need to contact you to undertake the study or audit the data collection process. The people who analyse the information will not be able to identify you and will not be able to find out your name, NHS number or contact details.
London North West Healthcare University NHS Trust will keep identifiable information about you from this study for 5 years after the study has finished.

11. **What happens if there is a problem?**

You will be monitored very closely in the post operative period whilst you are in hospital. The Research team will visit you to ensure you are happy with the device and understand how to use it. If there is any indication that there is a problem we will stop the trial and assess the situation fully. If the problem is resolved and you are happy to continue you may re-enter the trial. If you have a problem with the device out the hospital please stop using the device and contact the research team on the details below who will respond and provide any assistance or advice as necessary.

If there is a medical problem related to your surgery or underlying disease please either contact your clinical team, your GP or in an emergency the emergency services, unfortunately the research team, although clinicians are unable to advise on your general medical care.

If you have a complaint or grievance regarding any aspect of your care or participation in research which you would like to raise outside your clinical or research please contact the hospitals Patient Advice and Liason Service (PALS) on 0208 8695 653.

12. **What will happen if I don't want to carry on with the study?**

You may withdraw from participation in the project at any time and without any disadvantage to yourself. If you do withdraw, all information obtained until your withdrawal will be kept for analysis unless you ask for it to be withdrawn. You will not be able to withdraw after the study is complete.

13. **What are the possible benefits of taking part?**

Previous research has suggested that increased muscle mass leads to better outcomes after surgery for bowel cancer. There is evidence that NMES increases muscle mass. The aim of this study is to see if active treatment with NMES by mimicking exercise increases your muscle mass and as a result it improves outcomes such as fewer post operative complications, better quality of life and longer survival.

14. **Who is organising and funding the research and where was it reviewed?**

The study has been organised by Mr Jenkins (Consultant Surgeon and lead for complex colorectal cancer) and his team at St Mark's Hospital, Northwick Park Hospital, The University

of Northumbria and Imperial College London. This study also forms part of an educational project at Imperial College London supervised by Mr Jenkins. This study has been funded by a generous private donation to the St Mark's Hospital Foundation (registered charity number 1140930) explicitly for the BiCyCLE (Body Composition Manipulation in Colorectal Cancer) trial group at St Mark's and Imperial College London headed by Mr Jenkins.

15. Who has reviewed the research?

All research in the NHS is looked at by independent group of people called a Research Ethics Committee (REC), the purpose of which is to protect your interests. Prior to REC approval, this trial was reviewed and approved by an independent expert in functional electrical stimulation and an independent expert in colorectal surgery. Additionally, London North West University Healthcare NHS Trust Research and Development department have reviewed and given approval for the study to take place at St Mark's Hospital.

16. What are the possible side effects of using Mircostim?

The MicroStim 2v2 is used on a daily basis in clinical practice in the UK and has been deemed safe however with every medical intervention there are potential risks, which we are obliged to tell you about however unlikely they may be.

There is a potential risk that handling the electrodes while the stimulator is on can lead to stimulation current affecting other areas of the body. There is a theoretical possibility that the electrical stimulation may affect the heart if the current path is directly across the chest, although there are no reported incidences of this. To avoid this please always remember to turn off the stimulator before you remove the electrodes.

A slight reddening of the skin under the electrode is normal. This should fade after about an hour once the electrodes are removed. If stimulation causes long term marking of the skin, discontinue use and seek medical advice.

The following risks should not be relevant to you as part of our patient population but we are obliged to inform you of them and if you have any questions or concerns please ask. If you suffer from spastic tone in any of your muscles and if you notice any adverse change in the spasticity, discontinue use and seek medical advice. The safety of electrical stimulation in pregnancy has not been determined. Some high level spinal cord lesion patients may suffer autonomic effects after or during electrical stimulation. If headaches or sweating occur, or if blood pressure, bowel or bladder are affected, discontinue the use of the stimulator and seek medical advice.

17. Do's and Don'ts of the MS2v2 NMES

Do wash and dry the skin carefully once the electrodes have been removed.

Do remember to turn off the stimulator before you remove the electrodes.

Do clean the device using a damp cloth as required.

Do NOT use skin creams near the electrode sites.

Do NOT immerse the device in water. Instead,

Do NOT use spirit-based cleaners unless required for infection control reasons.

Do NOT place electrodes over broken skin or shave the area under the electrodes as this may cause skin irritation.

Do NOT operate dangerous machinery or drive whilst using the stimulator.

Do NOT use the electrical stimulation device in pregnancy.

Do NOT use if you have an implanted electronic device (pacemaker etc.)

Do NOT use the device if you suffer from epilepsy unless your fits are well controlled with drug treatment (please inform the research team if this is the case).

18. Who can I contact for further information?

If you have any questions now or in the future, please feel free to contact either:

**Colorectal cancer**<br>**Research Coordinator**<br>**R&D, Northwick Park, London North West University Healthcare NHS Trust**	Contact phone number:<br>020 8869 5088
**Mr Edward T Pring**<br>**Principle Investigator, George Davies Surgical Research Fellow & Registrar in General and Colorectal Surgery,**<br>**Imperial College London & St Mark's Hospital, London North West University Healthcare NHS Trust**	Contact phone number:<br>020 8869 4177<br>e.pring17@imperial.ac.uk
**Mr John T Jenkins**<br>**Chief Investigator, Consultant Colorectal Surgeon and Lead for Complex Cancer,**<br>**St Mark's Hospital, London North West University Healthcare NHS Trust**	Contact phone number:<br>020 8869 4177<br>i.jenkins@nhs.net
**Complex Colorectal Cancer Specialist Nurses**<br>**Claire Taylor**<br>**Macmillan Nurse Consultant**<br>**Therese Hona**<br>**Clinical Nurse Specialist**<br>**Complex Cancer Clinic**<br>**St Mark's Hospital, London North West University Healthcare NHS Trust**	Contact phone number:<br>0208 869 2487<br>Claire.taylor8@nhs.net<br>Therese.hona@nhs.net

This study has been approved by the London Queen's Square Research Ethics Committee.

Imperial College London

ST MARK'S HOSPITAL

BiCyCLE

Diagnosis of Primary or Recurrent Locally advanced rectal cancer — Week zero

Assessed for eligibility — Week one

Consent for participation

Pre operative baseline measures – Questionnaire, Berg Balance Scale, Sit-to-stand, 6 minute walk test, thigh circumference, BIA — Week 1-2

Randomisation

Preoperative treatment protocol	Placebo NMES protocol, patient
Radical Surgery	Radical Surgery
Postoperative treatment NMES	Placebo NMES protocol, patient

Week 2-4

Day of Surgery

Week 4-

Postoperative measures:

- Device feedback & Quality of Life Questionnaires 6 and 12 months
- Post-operative daily inflammatory markers
- Short term outcomes 30 day mortality
- In hospital/30 day morbidity Clavien-Dindo Classification
- Thigh circumference (bilateral) bio-impedance analysis
- 6 minute walk test, sit to stand and Berg Balance Scale
- Standard 5-year routine follow up protocol including CT Scans & Blood Tests including inflammatory markers.

Week 4 – 58 End of

Standard follow-

The BiCyCLe NMES Trial: A study to examine the effects of neuro-muscular electrical stimulation on body composition following major surgery for locally advanced rectal cancer

Chief Investigator: Mr J T Jenkins

Principle Investigator: Mr E T Pring

CONSENT FORM

Boxes will need to be initialled to confirm consent:

1.	I have read the information sheet (vers……) and consent form (vers……) concerning this study and understand the aims of this research project.	
2.	I have had sufficient time to talk with other people of my choice about participating in the study.	
3.	I confirm that I meet the criteria for participation which are explained in the information sheet.	
4.	All my questions about the project have been answered to my satisfaction, and I understand that I am free to request further information at any stage.	
5.	I know that my participation in the project is entirely voluntary, and that I am free to withdraw from the project at any time without giving any reason, without my medical care or legal rights being affected.	
6.	I understand that if I withdraw, all information collected until my withdrawal will be kept by the investigators.	
7.	I know that as a participant I will allow the researchers to access my medical records including CT scans performed at other NHS trusts which have been transferred to St Mark's Hospital/London North West University Healthcare NHS Trust for the purpose of my care and treatment and will be requested to complete questionnaires about my quality of life/experience of NMES.	
8.	I know that the questionnaire will measure my quality of life and that if the line of questioning develops in such a way that I feel hesitant or uncomfortable I may decline to answer any particular question(s), and /or may withdraw from the project without disadvantage of any kind.	

9.	I understand time associated with the use of and the potential risk of discomfort or harm from the use of the Neuro-Muscular Electrical Stimulator (NMES) has explained including pain, local skin irritation and reaction to the adhesive gel as listed in the Information Sheet.	
10.	I know that when the project is completed all personal identifying information will be removed from the paper records and electronic files which represent the data from the project, and that these will be placed in secure storage and kept for at least five years.	
11.	I agree for the chief investigator, if appropriate, to contact my general practitioner, consultant surgeon and colorectal specialist nurse to make them aware of my participation in the study.	
12.	I agree for the research team to contact my medical team at my local hospital to request and use my patient data which is relevant and applicable to this study including the transfer of CT scan images to St Mark's Hospital.	
13.	I agree to and understand that any written statements I make in response to research related questions within the questionnaires may be quoted in the final published study results	

Signature of Participant: Date:

Name of Participant:

Date:

Signature of Researcher:

Name of Researcher taking consent:

Three copies of this consent form should be signed: one to be kept in the patient notes, one to be given to the patient and one for the master trial folder.
